# Optical Coherence Tomography: An Eye Into the Coronary Artery

**DOI:** 10.3389/fcvm.2022.854554

**Published:** 2022-05-11

**Authors:** Ankush Gupta, Abhinav Shrivastava, Rajesh Vijayvergiya, Sanya Chhikara, Rajat Datta, Atiya Aziz, Daulat Singh Meena, Ranjit Kumar Nath, J. Ratheesh Kumar

**Affiliations:** ^1^Department of Cardiology, Military Hospital Jaipur, Jaipur, India; ^2^Department of Cardiology, Dr Ram Manohar Lohia (RML) Hospital & Atal Bihari Vajpayee Institute of Medical Sciences (ABVIMS), New Delhi, India; ^3^Advanced Cardiac Centre, Post Graduate Institute of Medical Education and Research (PGIMER), Chandigarh, India; ^4^University of Minnesota Medical Center, Minneapolis, MN, United States; ^5^Director General Armed Forces Medical Services, O/o DGAFMS, Ministry of Defence, New Delhi, India; ^6^Department of Cardiology, Jawahar Lal Nehru (J.L.N.) Medical College, Ajmer, India; ^7^Army Hospital Research and Referral (AHR&R), New Delhi, India

**Keywords:** OCT, IVUS, plaque morphology, saline OCT, calcified lesion modification, OCT in ACS, OCT in left main bifurcation angioplasty, OCT in bifurcation angioplasty

## Abstract

Optical coherence tomography (OCT) is slowly but surely gaining a foothold in the hands of interventional cardiologists. Intraluminal and transmural contents of the coronary arteries are no longer elusive to the cardiologist's probing eye. Although the graduation of an interventionalist in imaging techniques right from naked eye angiographies to ultrasound-based coronary sonographies to the modern light-based OCT has been slow, with the increasing regularity of complex coronary cases in practice, such a transition is inevitable. Although intravascular ultrasound (IVUS) due to its robust clinical data has been the preferred imaging modality in recent years, OCT provides a distinct upgrade over it in many imaging and procedural aspects. Better image resolution, accurate estimation of the calcified lesion, and better evaluation of acute and chronic stent failure are the distinct advantages of OCT over IVUS. Despite the obvious imaging advantages of OCT, its clinical impact remains subdued. However, upcoming newer trials and data have been encouraging for expanding the use of OCT to wider indications in clinical utility. During percutaneous coronary intervention (PCI), OCT provides the detailed information (dissection, tissue prolapse, thrombi, and incomplete stent apposition) required for optimal stent deployment, which is the key to successfully reducing the major adverse cardiovascular event (MACE) and stent-related morbidities. The increasing use of OCT in complex bifurcation stenting involving the left main (LM) is being studied. Also, the traditional pitfalls of OCT, such as additional contrast load for image acquisition and stenting involving the ostial and proximal LM, have also been overcome recently. In this review, we discuss the interpretation of OCT images and its clinical impact on the outcome of procedures along with current barriers to its use and newer paradigms in which OCT is starting to become a promising tool for the interventionalist and what can be expected for the immediate future in the imaging world.

## Introduction

Recently, intravascular imaging has become an integral part of coronary intervention. Intracoronary imaging using intravascular ultrasound (IVUS) and optical coherence tomography (OCT) empowers the interventionalist with simultaneous cross-sectional views of the coronary artery, which compliments conventional longitudinal coronary artery angiography. The “lumenogram” obtained after injecting a radiopaque contrast medium inside the coronary artery under fluoroscopy gives a planar projection of the intracoronary anatomy open to visual interpretation (1–3). On one hand, it is an excellent modality to assess the severity of stenosis, albeit with high interobserver variability. However, it is also accompanied by several limitations, such as vessel overlap, foreshortening, poor resolution, and angle dependence, and is particularly deficient when it comes to imaging of the vessel wall itself regarding the amount of atherosclerosis, circumferential involvement, and lesion depth and composition (1). The limitations of coronary angiography are largely mitigated by the recreation and analysis of intramural and transmural coronary artery anatomy with the aid of intracoronary imaging modalities (4). Indeed, the latest European Society of Cardiology (ESC)/EACTS guidelines updated an indication for the use of OCT for stent optimization to a class IIa recommendation, which corresponds to the same level of recommendation as IVUS (5). Both imaging modalities have shown to be superior to conventional angiography for the optimization of percutaneous coronary intervention (PCI), the higher resolution provided by OCT offers a more detailed assessment of plaque morphology, histopathologic features (macrophages, coronary vasa vasorum, and cholesterol crystals), stent deployment parameters, and stent failure. Table 1 shows the differences between IVUS and OCT.

**Table 1:** Comparison between intravascular ultrasound (IVUS) and optical coherence tomography (OCT).

**Parameter**	**IVUS**	**OCT**
**Bio-Photonics**		
Wave source	Ultrasound	Light
Wave length	40,000 nm	1,250 to 1,350 nm
Axial resolution	50 – 150 μm	10 – 20 μm
Penetration depth	5-6 mm	1-3 mm
Blood clearance	Moderate backscatter from blood. Does not require blood clearance	Requires clearance of blood
**Pre-PCI lesion assessment**		
Visualization of intima (OCT superior)	++	+++
Visualization of EEL under plaque burden (IVUS superior)	+++	+
Plaque microstructures (OCT superior)	+	+++
Plaque calcium (OCT superior)	++	+++
Plaque vulnerability (OCT superior)	+	+++
Thrombus (OCT superior)	+	+++
**Post PCI optimization**		
Stent malapposition (OCT superior)	++	+++
Stent expansion (Equivalent)	+++	+++
Plaque prolapse (OCT superior)	++	+++
Stent edge dissection (OCT superior)	++	+++
Left main and ostial disease (IVUS superior)	+++	+
Assessment of stent failure (OCT superior)	++	+++
Biodegradable stents (OCT superior)	+	+++

*+ its feasible, ++ its good, +++ its an excellent imaging modality for that particular subset*.

In this review, we attempt to describe the current evidence and clinical applications of OCT in guiding PCI, including the assessment of plaque morphology, selection of appropriate devices for plaque modification, and optimization after stenting. We also discuss the traditional pitfalls of OCT along with current barriers to its use and the newer paradigms in which OCT is starting to become a promising tool for the interventionalist and what to expect for the immediate future in the imaging world.

## History Of Oct

Naohiro Tanno of Japan and James G. Fujimoto of the USA first studied OCT independently and patented it almost simultaneously (6). After *in vitro* studies of the retina and coronary arteries in 1991, this technology was applied clinically in ophthalmology from 1996 onward. A new era of intracoronary imaging began in 2002 when the first clinical study of OCT was conducted (7). One of the initial challenges to the use of OCT in the coronary arteries was the need to clear the blood field for imaging. This was initially achieved with sustained low-pressure balloon occlusion in time domain (TD) OCT, but has recently been entirely replaced by a faster, newer generation frequency domain (FD) OCT systems, which utilize viscous contrast as a flushing media for coronary image acquisition. Coronary OCT (TD) was first introduced to the market in Europe in 2007 and, after FDA approval, in the USA in 2010. Since then, its use has grown very rapidly.

## Clinical Adaptation Of Oct

The feasibility of OCT (TD) in intracoronary imaging was first established in 2002 by a study comparing its images to those obtained by IVUS (7). In 2007, Prati et al. established the safety of a non-occlusive technique using OCT in 64 patients, with a success rate of around 94% (8). With the simplification of the image acquisition process, the new generation FD-OCT system provided faster and more reliable images of the coronary artery with good image reproducibility and no significant procedure-related adverse events (9). Imaola et al. established the safety and feasibility of FD-OCT in 2010 (10). A few complications encountered were all resolved before the patient left the catheterization laboratory. Further large-scale registries have demonstrated that intra-procedural complications with OCT are very rare (all <0.2%) and similar to the event rates that occur during IVUS image acquisition (11).

Optical coherence tomography has distinct advantages over angiographic guidance, both subjectively and in major trials over many years (Table 2). Studies have shown improved survival and reduced major adverse cardiovascular events (MACEs) in patients undergoing OCT-guided PCI optimization compared with angiography only (12, 18). OCT significantly changed operator behavior during PCI planning and after stent placement (15, 17, 20). OCT-guided PCI optimization resulted in significantly higher post-PCI fractional flow reserve (FFR) (15, 16), stent strut coverage (14), and stent expansion (17, 19) compared with angiography-guided PCI. Compared with IVUS (Table 3), OCT-guided PCI initially resulted in smaller minimum stent area (MSA), stent expansions (21), and stent diameters, albeit with similar rates of target vessel failure (TVF) and restenosis in a 12-month follow-up (24). As part of the evolution of OCT, the stent sizing protocol evolved from initial lumen sizes to reference external elastic lamina- (EEL-) based sizes resulting in MSA and stent expansion comparable to those of IVUS (17, 19, 25). Phantom model trials showed that the mean lumen area measured by OCT was equal to the actual lumen area of the phantom model while IVUS overestimated it (22). OCT was also found to be more sensitive in detecting intrastent tissue protrusion, incomplete stent apposition, stent edge dissection, and intrastent thrombus compared to IVUS (17, 19, 22).

**Table 2:** Studies performed for a comparison between OCT and angiography for percutaneous coronary intervention (PCI) optimization.

**Study/Journal/Year/Design**	**Sample size**	**Primary end point**	**Result**	**Conclusion**
CLI-OPCI (12)/Euro-Intervention/2012/ Retrospective, multicenter	• *N* = 670 • 335 in the OCT group • 335 in the Angio group	1-year rate of cardiac death or MI	• OCT group vs. angio group- - Cardiac death (1.2 vs. 4.5%, *p* = 0.010) - Cardiac death or MI (6.6 vs. 13.0%, *p* = 0.006) - Composite of cardiac death, MI, or repeat revascularization (9.6 vs. 14.8%, *p* = 0.044) - Post PCI OCT in the OCT group revealed adverse features requiring further interventions in 34.7% patients (1/3rd)	OCT guided optimization can improve clinical outcomes of patients undergoing PCI
CLI-OPCI II (13) (JACC Cardiovasc Imaging)/2015/ Retrospective analysis, multicenter	1,002 lesions (832 patients)	• 1-year MACE (composite of all-cause mortality, MI and TLR) • Association of sub-optimal stent deployment as assessed by offline OCT with MACE at 1 yr	• Sub-optimal stent deployment required the presence of at least 1 of the OCT findings- 1. Edge dissection: Presence of a linear rim of tissue with clear separation and a width >200 mm, (<5 mm) to a stent edge 2. Malapposition: stent-adjacent vessel lumen distance >200 mm 3. In-stent minimum lumen area (MLA) <4.5 mm^2^ 4. In-stent MLA <70% of the average reference lumen area 5. Intrastent plaque/thrombus protrusion >500 mm in thickness • Suboptimal stent seen in 31.0% patients • Independent predictors of MACE were In-stent MLA <4.5 mm2 (*p*−0.040), dissection >200 mm at the distal stent edge (*p*−0.004), and reference lumen area <4.5 mm^2^ at either distal (*p* < 0.001) or proximal (*p* < 0.001) stent edges	- Suboptimal stent deployment was associated with an increased risk of MACE - Presence of at least 1 significant criterion for suboptimal OCT stent deployment was confirmed as an independent predictor of MACE (HR: 3.53, *p* < 0.001) - MACE group had significantly more findings of sub-optimal stent deployment (59.2 vs. 26.9%; *p* < 0.001)
OCTACS study (14)/Circulation: Cardiovascular interventions/2015/RCT, Single center	• 100 patients • 1:1 • OCT-guided vs angio-guided Nobori biolimus-eluting DES implantation	Difference in percentage of uncovered struts in the OCT-guided vs. the angio-guided group at 6-months	OCT-guided PCI resulted in a lower proportion of uncovered struts (4.3 vs. 9.0%, *P* < 0.01) and more number of completely covered stents (17.5 vs. 2.2%, *P* = 0.02)	OCT-guided optimization of DES improves strut coverage in comparison with angiographic guidance alone
ILUMIEN I (15)/European Heart Journal/2015/Prospective, non-randomized, observational	418 patients (467 stenosis)	• Impact of OCT on physician decision-making • MACE at 30 days (cardiac death, MI and target lesion revascularization)	• Pre-PCI OCT - Altered strategy in 55% of patients (57% stenosis) - Selecting different stent lengths shorter in 25%, longer in 43% • Post PCI OCT - Unsatisfactory result in 25% of patients (27% stenosis) - 14.5% malapposition - 7.6% under-expansion - 2.7% edge dissection	- Decision-making was affected by OCT imaging prior to PCI in 55% and post-PCI in 25% patients - MACE events at 30 days were low: death 0.25%, MI 7.7%, repeat PCI 1.7%, and stent thrombosis 0.25%
DOCTORS (16)/Circulation/2016/ RCT, multicenter	• *N* = 240 (NSTEMI-ACS) • 120 in the OCT group • 120 in the Angio group	FFR post PCI	• Significantly higher FFR in OCT group (0.94 ± 0.04 vs. 0.92 ± 0.05, *P* = 0.005) compared to angiographic guided group • OCT led to altered procedural strategy in 55% patients • Post-PCI OCT revealed - Stent under-expansion 42% - Stent malapposition 32% - Incomplete lesion coverage in 20% - Edge dissection in 37.5% • Led to the more frequent use of poststent dilation in the OCT-guided group vs. the angiography-guided group (43 vs. 12.5%, *P* < 0.0001) with lower residual stenosis (7.0 vs. 8.7%, *P* = 0.01)	• OCT-guided PCI is associated with higher post procedure FFR • No significant difference in the rate of procedural MI or complications
ILUMIEN III (17)/Lancet/2016/RCT, multicenter	• *N* = 450 • 158 [35%] in OCT • 146 [32%] in IVUS • 146 [32%] angiographic guidance • Exclusion—LM or ostial RCA, bypass graft stenosis, CTO, planned two-stent bifurcations, and ISR	• Post-PCI MSA as assessed by OCT • Tested- - Non-inferiority of OCT guidance to IVUS guidance -Superiority of OCT guidance to angiography guidance - Superiority of OCT guidance to IVUS guidance	- Final median MSA was 5.79 mm^2^ with OCT and 5.49 mm^2^ with angiography guidance. OCT guidance was not superior to angiography guidance (*p* = 0.12) - Minimum and mean stent expansion was significantly improved by OCT [87.6% and 105.8%] as compared with angiography (82.9%, *P* = 0.02 and 101.4%, *P* = 0.001, respectively) - Untreated major dissections at end of procedure and major malapposition were significantly less in OCT guided group (28 and 11%) compared with angiography group (44%, *P* = 0.006 and 31%, *P* < 0.0001)	• OCT guided stent placement was not superior to angiography-guided stent placement in terms of MSA • Minimum and mean stent expansion were significantly greater while untreated major dissections and major malapposition were significantly less in OCT than with angiography-guided PCI
LONDON PCI COHORT (18)/JACC: Cardiovascular interventions/2018/ Registry (Observational)	OCT in 1,149 (1.3%) patients, IVUS in 10,971 (12.6%) patients Angiography alone in 75,046 patients	All-cause mortality at a median of 4.8 years	OCT-guided PCI was associated with significantly reduced mortality rates when compared with angiography alone (9.60 vs. 16.80%; *p* < 0.0001)	OCT-guided PCI was associated with improved MACE and long-term survival compared with angiography-guided PCI
iSIGHT (19)/Circulation: Cardiovascular interventions/2021/RCT	• *N* = 156 lesions • OCT [51 lesions (32.7%)] • IVUS [52 lesions (33.3%)] Angiography [53 lesions (34.0%)]	Stent expansion (MSA ≥ 90% of the average reference lumen area)	Stent expansion with OCT guidance (98.01 ± 16.14%) was superior to angiography (90.53 ± 14.84%, *P* = 0.041)	Stent expansion with OCT guidance was superior to an optimized angiographic strategy

**Table 3:** Studies performed for a comparison between OCT and IVUS for PCI optimization.

**Study/Journal/Year/Design**	**Sample size**	**Primary end point**	**Stent sizing criteria in OCT guidance**	**Result**	**Conclusion**
Habara et al. (21)/Circulation: Cardiovascular interventions/2012/ RCT, single center	• *N* = 70 • OCT group *n* = 35 IVUS group *n* = 35	Stent expansion analyzed by IVUS	Lumen diameter	- Focal and diffuse stent expansion were smaller (64.7 vs. 80.3%, 84.2 vs. 98.8%, *P* < 0.05, respectively), in OCT vs IVUS group - Minimum and Mean stent area was smaller (6.1 vs. 7.1 mm^2^ & 7.5 ± 2.5 vs. 8.7 ± 2.4 mm) under OCT vs. IVUS	OCT guidance for stent implantation was associated with smaller stent expansion compared with conventional IVUS guidance
OPUS-CLASS (22)/JACC: Cardiovascular Imaging/2013/ Prospective, multicenter	• *N* = 100 • Angiography performed in all • Followed by OCT and IVUS in - Pre-PCI−20 - Pre-& Post PCI-60 - Post PCI follow-up −20	Comparison of lumen dimensions measurement with OCT vs. IVUS vs. angiography (QCA) in patient and in phantom model	Lumen diameter	- Mean minimum lumen diameter measured by QCA was significantly smaller than that measured by FD-OCT (1.81 mm^2^ vs. 1.91 mm; *p* < 0.001) - The minimum lumen area measured by IVUS was significantly greater than that by FD-OCT (3.68 vs. 3.27 mm^2^; *p* < 0.001) - In a phantom model, the mean lumen area according to OCT was equal to the actual lumen area of the phantom model; IVUS overestimated the lumen area	• OCT provided accurate measurements of coronary lumen with excellent intra-observer reproducibility • OCT is much more sensitive in detecting intrastent tissue protrusion, incomplete stent apposition, stent edge dissection, and intrastent thrombus compared with IVUS
ILUMIEN II (23)/JACC: Cardiovascular interventions/2015/ *Post-hoc* analysis of 2 prospective studies	OCT-guided stenting in patients in the ILUMIEN I, *N* = 354 vs. IVUS-guided stenting in the ADAPT-DES, *N* = 586	Post-PCI stent expansion (%) defined as the MSA divided by the mean reference lumen area	Lumen diameter	Degree of stent expansion was not significantly different between OCT and IVUS guidance 72.8 vs. 70.6%, respectively, *p*−0.29	OCT and IVUS guidance resulted in a comparable degree of stent expansion
OPINION (24)/European Heart Journal/2017/RCT, multicenter, non-inferiority trial	• *N* = 829 • OCT guided PCI = 414 • IVUS guided PCI *n* = 415	Target vessel failure (TVF) defined as a composite of cardiac death, MI, and ischemia-driven target vessel revascularization until 12 months after the PCI	Lumen diameter	- TVF occurred in 21 (5.2%) undergoing OCT-guided PCI and 19 (4.9%) undergoing IVUS-guided PCI (non-inferiority = 0.042) - Rate of binary restenosis was comparable between OCT-guided PCI and IVUS-guided PCI (in-stent: 1.6 vs. 1.6%, *P* = 1.00; and in-segment: 6.2 vs. 6.0%, *P* = 1.00)	OCT-guided PCI was non-inferior to that of patients undergoing IVUS-guided PCI Stent sizes were smaller in the OCT arm compared with the IVUS arm (2.92 vs. 2.99 mm; *p* = 0.005)
ILUMIEN III/(17)/Lancet/2016/ RCT, multicenter	• *N* = 450 • 158 [35%] in OCT • 146 [32%] in IVUS • 146 [32%] angiographic guidance	Post-PCI MSA as assessed by OCT	Proximal or distal normal segment EEL diameter (whichever is lesser) rounded down to the nearest 0.25mm	- Final median MSA was 5.79 mm^2^ with OCT and 5.89 mm^2^ with IVUS guidance - OCT guidance was non-inferior to IVUS guidance (*p* = 0.001), but not superior (*p* = 0.42) - Untreated dissections and major malapposition were significantly less frequent in OCT group compared with IVUS group	OCT-guided PCI using reference segment EEL-based stent optimization strategy was safe and resulted in similar MSA to that of IVUS-guided PCI
MISTIC-1 (25)/Circulation: Cardiovascular Interventions/2020/ RCT, multicenter, non-inferiority trial	• *N* = 109 • OCT- 54 patients with 62 lesions • IVUS-55 patients with 64 lesions	In-segment MLA assessed using OCT at the 8-month follow-up	Lumen up-size for OCT guidance (10% or 0.25-mm larger than mean lumen diameter at reference sites)	• Post-procedural minimum stent area was 6.31 mm^2^ and 6.72 ± 2.08 mm^2^ in OCT and IVUS group, respectively (*P* = 0.26) • 8-month follow-up, in-segment MLA was 4.56 & 4.13 mm^2^ in OCT and IVUS group, respectively (non-inferiority <0.001)	OCT-guided PCI was not inferior to IVUS-guided PCI in terms of in-segment MLA at 8 months Clinical outcomes at 3 years follow-up did not differ between the two groups
iSIGHT (19)/Circulation: Cardiovascular Interventions/2021/ RCT	• *N* = 156 lesions • OCT [51 lesions (32.7%)] • IVUS [52 lesions (33.3%)] Angiography [53 lesions (34.0%)]	Stent expansion (MSA ≥ 90% of the average reference lumen area)	• When EEL was visible in ≥180° of the vessel circumference, the reference was sized to the mean EEL diameter • Otherwise, the largest lumen diameter was used	Stent expansion with OCT guidance (98.01 ± 16.14%) was noninferior to IVUS (91.69 ± 15.75%, non-inferiority <0.001)	Stent expansion with OCT guidance using a dedicated EEL–based sizing strategy was non-inferior to that achieved with IVUS

## Image Acquisition With Oct

Many established companies have made OCT imaging systems commercially available over the years; however, the two most widely available and employed systems are the OPTIS™ system (Abbott Vascular, Santa Clara, CA, USA) with angiographic and OCT visualization (co-registration) options and the Lunawave^®^ system (Terumo, Tokyo, Japan). A typical OCT imaging system consists of a catheter, a motor, and an imaging console with software. The guide catheter is engaged at the coronary ostium, and the OCT catheter is advanced on the guidewire with the imaging lens placed distal to the area of interest. The guide catheter is then flushed with contrast to clear the blood. The amount and pressure of the contrast injection required for blood clearance depend on the coronary system being imaged (right or left), its size and length, and the location of the area of interest (proximal or distal). Usually, 14–16 ml contrast is sufficient for the left coronary artery at a rate of 4 ml/s, and 10–12 ml contrast is sufficient for the right coronary artery (RCA) at a rate of 3 ml/sec. Contrast may be hand injected or flushed with an automated injector. During manual injection, it is imperative to monitor the OCT console to guide the pressure and volume of contrast injection. As soon as the guide catheter is seen on the pullback, contrast injection should be stopped to minimize contrast load. Pullbacks of 75- and 54-mm in length are available with the current OCT system. The 75-mm pullback is faster, requires less contrast, captures 5 frames per mm of pullback, and is usually sufficient to study plaque morphology and coronary dimension measurement prior to stent placement. Because the 54-mm pullback is slow, it requires more contrast for image acquisition and provides 10 frames per mm of pullback, thereby giving a resolution better than 75 mm. It renders high-quality images of the stent after deployment as well as complications such as stent fracture. The 54-mm pullback is also useful to guide the position of the guide wire recrossing in the side branch (SB) during bifurcation angioplasty. While imaging the distal coronaries with OCT, it is essential to ensure that the catheter does not get railed off from the guidewire as it can get stuck in the distal stent struts, making retrieval difficult (26).

## Oct For Pci Optimization

Intracoronary OCT provides a detailed characterization of the arterial wall and lumen, which is complimentary to angiography. It plays a key role in the pre-PCI assessment of plaque morphology to choose an appropriate plaque modification device. Lesion length and coronary diameter can be accurately measured using EEL to guide the required stent size. Post-PCI optimization includes the assessment of stent apposition, stent expansion, significant edge dissection, geographical miss, and plaque prolapse.

### Pre-PCI Assessment

#### Plaque Morphology

Interpretation of OCT images as normal and pathological ones requires an understanding of the optic attenuation characteristics of the vascular layers. When the light rays are directed at a tissue, some portion of them are able to pass through and some get scattered or reflected. The amount of backscatter determines the brightness of the tissue. The amount of light absorbed by the tissue during its passage is known as tissue attenuation, which determines the penetration depth of OCT.

**Figure 1:**
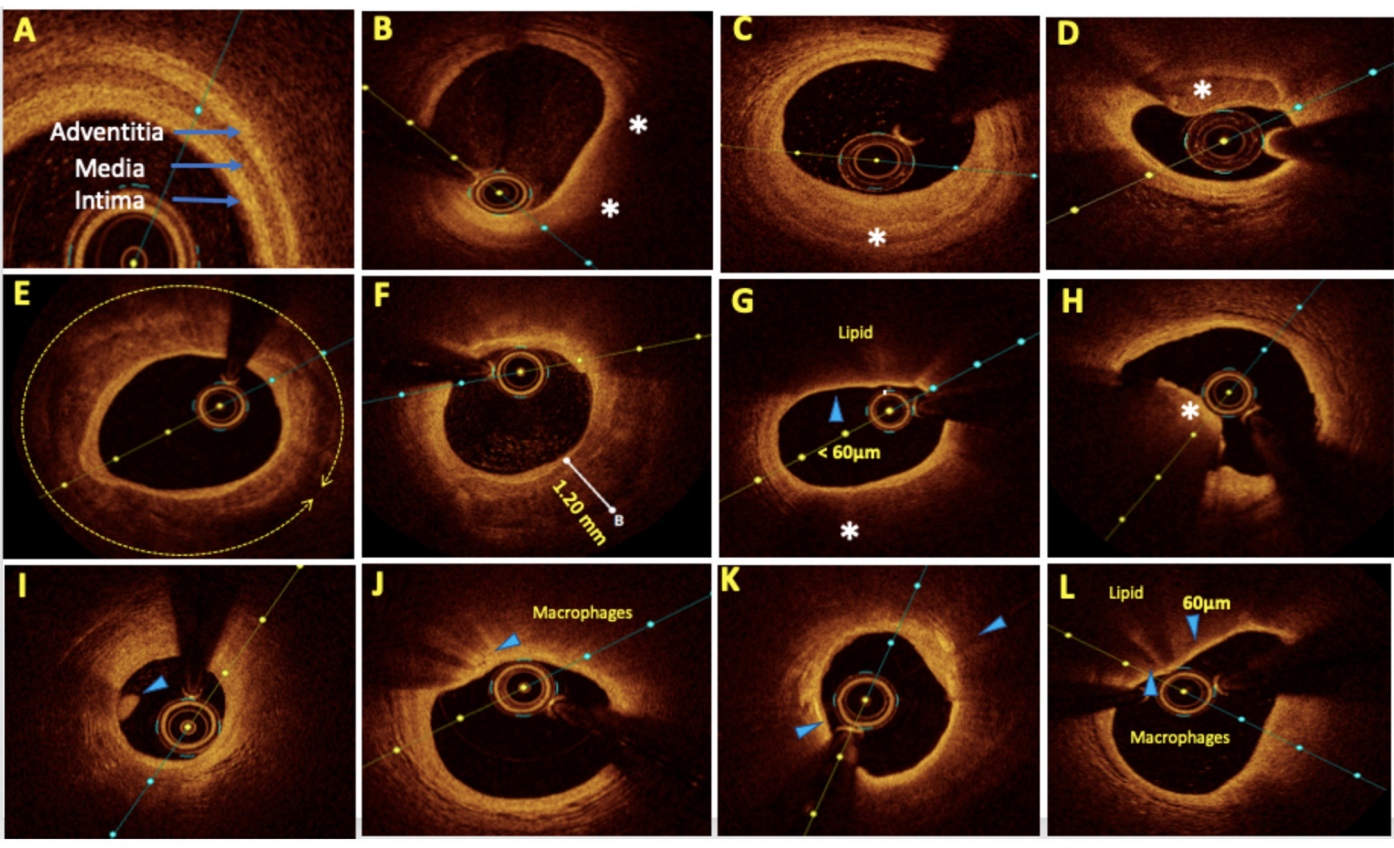
Representative optical coherence tomography (OCT) images with various plaque morphologies. **(A)** Normal coronary, **(B)** lipid-rich plaque (LRP), **(C)** fibrotic plaque, **(D)** calcific nodule, **(E)** near 360° arc of calcific plaque, **(F)** deep calcium deposition, **(G)** thin cap fibroatheroma (TCFA), **(H)** intraluminal red thrombus, **(I)** intraluminal white thrombus, **(J)** bright spots or bands at the boundary between the fibrous cap and lipid core suggestive of macrophages, **(K)** bright signal-rich cholesterol crystals, and **(L)** vulnerable plaque formed by a large lipid pool covered by TCFA with macrophage infiltration. Areas of interest were highlighted by arrows and asterisks.

The intima and adventitia have abundant collagen and elastin tissues that appear bright on the OCT image due to their intrinsic backscattering properties. In contrast, the medial layer possesses higher amounts of smooth muscle cells, which have poor reflective properties and thus appears dark on an OCT image. This “bright-dark-bright” pattern is characteristic of the trilaminar appearance of a normal coronary artery (Figure 1A) and the deposition of atherosclerotic plaques leads to the alteration of this pattern. The optical properties and OCT image interpretation of various atherosclerotic plaque morphologies have been described previously (27) and are summarized in Table 4.

**Table 4:** Showing optical properties and OCT image interpretation of various atherosclerotic plaque morphologies.

**Tissue**	**Optical properties**		**Imae**
	**Backscatter**	**Attenuation**	
Lipid	Low	High	Anatomic border with fibrous layer cannot be made out due to high backscattering. But then light attenuates much faster. So, lipid pool is progressively dark (Figure 1B)
Fibrous	High	Low	Bright signal rich homogenous appearance (Figure 1C)
Calcified tissue	Low	Low	Signal poor region with sharp boundaries between calcified and fibrous tissue (Figures 1D–F)
Thin cap fibrous atheromas (TCFA)	• Cap–High • Core–low	• Cap–Low • Core–High	Bright and signal rich fibrous cap followed by signal poor area of lipid pool with cap thickness <65 μm. TCFAs are more prone for plaque rupture (Figure 1G)
Red thrombus	High	High	Intraluminal mass and casts a shadow on the vessel walls due to high attenuation of RBCs (Figure 1H)
White thrombus	High	Low	Intraluminal mass with no attenuation (Figure 1I)
Macrophages	High	High	Bright spots or bands at border between fibrous cap and lipid core casting shadow behind it (Figure 1J)
Cholesterol crystal	High	Low	Thin & linear structure in the plaque (Figure 1K)

##### Plaque Modification as Per Morphology

**Figure 2:**
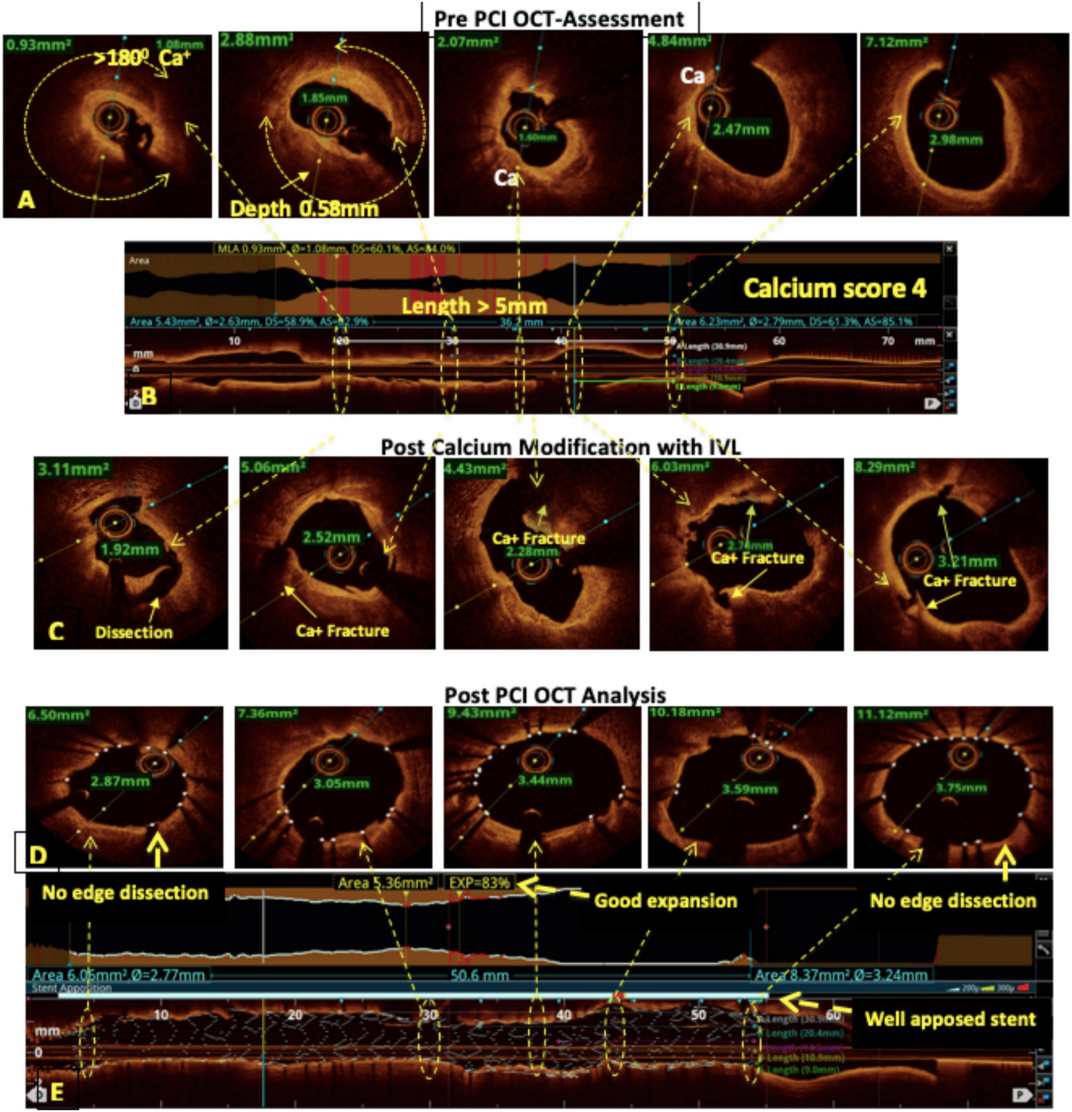
OCT-guided management of calcified left anterior descending (LAD). **(A,B)** Pre-percutaneous coronary intervention (PCI) assessment showed a heavily calcified LAD with a calcium arc >180°, a calcium thickness of 0.58 mm, and a length of >5 mm resulting in a calcium score of 4. **(C)** Fractures in the calcium after modification with intravascular lithotripsy (IVL). **(D,E)** Post-PCI assessment showed 83% stent expansion, no edge dissection, and a well-apposed stent.

- **Lipid-rich plaque (LRP)**: Lesions composed mainly of a necrotic lipid core with a fibrous cap are labeled as fibroatheromas. The thickness of the overlying cap determines its risk of rupture, and a thickness of <65 μm [thin cap fibroatheroma (TCFA)] has been seen to be particularly prone to rupture in pathologic series. Significant stenoses due to predominantly LRPs and TCFAs (Figures 1B,G) can be treated with direct stenting without predilatation.
- **Fibrotic plaque:** Fibrotic plaques are typically rich in collagen or smooth muscle cells and have a homogeneous signal-rich backscatter on OCT (Figure 1C). Primarily, fiber-rich plaques are mostly described as stable plaques and the likelihood of their rupture is proportional to the amount of internal lipid deposition. Primarily, fibrotic lesions that cause significant obstructive disease can be tackled with the use of cutting balloons or non-compliant balloons for adequate bed preparation prior to stent deployment to optimize stent expansion. Fibrofatty lesions with predominantly fatty composition require compliant balloons for bed preparation.
- **Vulnerable plaques:** The most vulnerable plaques can be recognized on OCT as having a large lipid core (lipid in ≥2 quadrants in any image) and a thin fibrous cap (<65 μm) (28). Stenosis caused by TCFAs with evidence of active inflammation, i.e., macrophage infiltration is seen as multiple punctate signal-rich regions near the fibrous cap and is a sign of increased vulnerability of a plaque (Figure 1L) (29–31). In 2019, the CLIMA study (32) defined a high-risk vulnerable plaque to be having four criteria, namely, (1) minimum lumen area (MLA) <3.5 mm^2^; (2) fibrous cap thickness of <75 μm at the thinnest portion; (3) lipid core with lipid arc extension of >180°; and (4) the presence of a cluster of macrophages within the plaque. The presence of these four features was found to be associated with a higher risk of major coronary events in a 1-year follow-up. A few studies have also shown adverse outcomes in diabetic patients with TCFAs, even in intermediate lesions with a negative FFR (33). The merits of stenting non-obstructive (50–70%) TCFA lesions have long been debated. The PROSPECT ABSORB pilot trial (34) nested within the PROSPECT II trial (35) suggested that PCI of plaques with high-risk “vulnerable” characteristics even with first-generation everolimus-eluting absorb bioresorbable vascular scaffolds (BVS) stent was safe and effective in enlarging the lumen and providing a cap of neointimal hyperplasia (NIH) with a trend toward reduced MACE rates in a 25-month follow-up. More data are needed to guide the management of intermediate vulnerable plaques, including the contribution of optimal medical therapy on plaque modification and regression as well as the role of prophylactic PCI.
- **Calcified lesion:** Calcified lesions causing significant stenosis are the most common cause of under-expanded stents (UESs), resulting in acute and chronic stent failure. OCT has a distinct advantage over IVUS for calcified lesion characterization and quantification. Calcium deposition in a lesion is classified into superficial, deep, and nodular calcium (Figures 1D–F). An OCT-based calcium score has been established and validated to determine the probability of stent under-expansion in calcified lesions (36). This “rule of 5s” score consists of three components, i.e., maximum thickness > 0.5 mm (1 point); contiguous length of calcium > 5 mm (1 point); and maximum arc > 50% or > 180° (2 points). A score of ≤ 3 resulted in acceptable stent expansion while a score of 4 had significantly lower stent expansion and indicated calcium modification during lesion preparation (Figure 2). Calcium fracture as clearly seen in OCT should be the target of modification as it leads to improved stent expansion compared to calcified lesions without fractures (37). A proposed algorithm for calcified lesion modification with devices according to their location and OCT calcium score is summarized in Table 5 although the same has not yet been validated.

**Table 5:** The calcified lesion modification devices according to its location and OCT calcium score.

**Calcified lesion**			**Strategy for lesion preparation**
Deep	Calcium score ≤ 3		NC/Cutting balloon/Scoring balloon
	Calcium score 4		Intravascular lithotripsy (IVL)
Superficial	Calcium score ≤ 3		NC/Cutting balloon/Scoring balloon
	Calcium score 4	Balloon crossable	IVL/Ultra-high-pressure balloon ± RA/OA
		Balloon non-crossable	RA or OA → ± IVL/Ultra-high-pressure balloon
Nodular			RA or OA

*NC, non-complaint; RA, rotational atherectomy; OA, orbital atherectomy*.

#### Lesion Length Estimation

The length is estimated using the L-mode view of the OCT pullback, which utilizes the pullback speed and frame rate for calculations. The catheter can be displaced during pullback due to the dynamic movement of the coronary arteries during the cardiac and respiratory cycles. However, the faster frame rates and speed of an FD-OCT system minimize this longitudinal catheter displacement as a potential source of error (38). In the L-mode view, the normal-appearing segments within 5 mm of the stenotic lesion, that is, segments with a preserved trilaminar morphology or minimal atherosclerotic distortion are selected as proximal and distal stent landing zones. In the case of diffusely diseased coronaries, segments with OCT markers of stable plaque-like thick fibrous cap atheroma and calcified plaque are chosen as landing zones. Visual confirmation of landing zones avoids stent edge problems, including stent length mismatches and landing on TCFAs, which leads to reduced edge dissections and also reduces the risk of early stent thrombosis and target lesion failure (13, 39–42).

#### Diameter Estimation

EEL-based measurements at the distal landing zone should be used for stent sizing. Two separate diameter measurements should be taken at least one quadrant apart, and the mean of these two measurements is rounded down by 0.25 mm to the available stent size (17). If the EEL is not visualized sufficiently, the mean lumen diameter is utilized for stent sizing and should be rounded up between 0.25 and 0.5 mm to determine the stent size (24).

#### Functional Assessment

The decision of whether or not to perform PCI in an intermediate lesion is another dilemma faced by cardiologists. Apart from the age-old practice of eyeballing from various angles and views, the use of FFR is regarded as the most valuable tool for decision-making in patients with intermediate lesions. As OCT is able to accurately characterize coronary lesions in a three-dimensional (3D) view, it may be used to assess the lesion functionally as well. Although FFR_OCT_ (computational FFR using the OCT data) showed good correlation and agreement with invasively measured FFR (*r* = 0.72, *p* < 0.001) (43), head-to-head comparisons between FFR and OCT for functional assessment of an intermediate lesion are very limited. The FORZA trial (44) compared FFR and OCT guidance in these patients. Under OCT guidance, PCI was performed in an intermediate lesion if at least one of the following criteria was present: (1) area stenosis (AS) >75%; (2) AS between 50 and 75% and MLA <2.5 mm^2^; and (3) AS between 50 and 75% and plaque rupture. In the FFR arm, PCI was performed when the lesion was <0.80 on FFR. The 13-month follow-up of the study showed that OCT was safe but led to more PCI procedures. However, it was ultimately associated with a lower occurrence of the combined endpoint of MACE or significant angina after 13 months. FFR resulted in more conservative management of the patient which was associated with less costs; however, it had a higher incidence of unplanned revascularizations. At present, studies comparing FFR and FFR_OCT_ are inconclusive and warrant further trials.

### OCT During Stent Deployment

The OCT angiography co-registration (ACR) feature, if available, is very useful in identifying stent edge landing zones and thus eliminating ambiguity in visually selecting “normal-appearing” reference segments on angiography. It provides a side-by-side visual correlation between OCT images, on which the planning can be done, with the real-time coronary angiogram on which the application of the plan is to be carried out (Supplementary Video 1). Schneider et al. (45) showed that major edge dissection and/or geographical miss was significantly reduced in ACR-guided PCI than in OCT-guided PCI without ACR [(4.2 vs. 19.1%), *p* = 0.03)]. Koyama et al. (46) revealed that the use of ACR resulted in a trend toward a reduced incidence of significant distal stent edge dissections (11.1 vs. 20.8%, *p* = 0.07). An operator vs. computer-based DOCTOR fusion study in 22 patients showed that, in the absence of co-registration, target lesion segments on OCT were uncovered by the stent in 14 patients (70%) and the mean “geographic miss distance” was 5.4 ± 2.6 mm (20). Stent placement in the SB can be optimized *via* the real-time ACR with the respective OCT cross-sectional image [“bifurcation and ostial OCT mapping” (BOOM)] (47) during bifurcation angioplasty. This technique can minimize the protrusion of stent struts into the main branch (MB) while ensuring full coverage of the SB ostium.

### Post-PCI Assessment

Optical coherence tomography-guided post-PCI stent optimization includes the estimation of stent expansion, stent apposition, significant edge dissection, plaque prolapse, and geographical miss.

#### Stent Expansion

**Figure 3:**
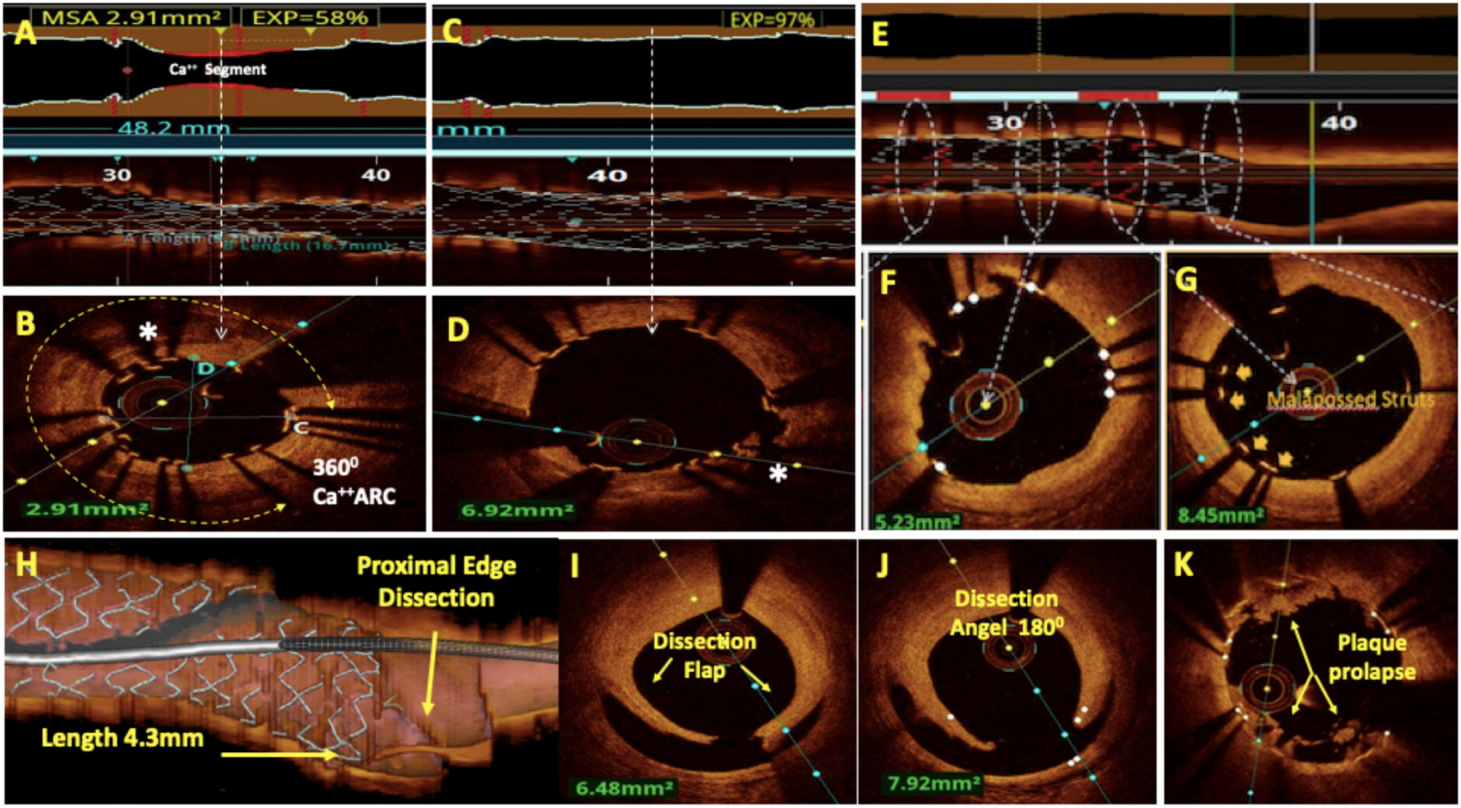
Post-PCI assessment by OCT. **(A)** Lumenogram and L mode showed under-expanded stent (UES) with minimum stent area (MSA) of 2.91 mm^2^ and 58% stent expansion. **(B)** Cross-sectional image at MSA revealed 360° arc of calcium as a cause of UES. **(C)** Lumenogram and L mode showed 97% stent expansion after the treatment of UES with ultra-high-pressure balloon dilatation. **(D)** A cross-sectional image shows the fracture of the calcium arc resulting in the resolution of the UES. **(E)** A rendered stent view shows well-apposed stent struts (white) and malapposed stent struts (red) corresponding to the white and red bars in the apposition indicator. Apposition **(F)** and mal-apposition **(G)** of the stent struts can also be well-appreciated on respective cross-sectional images. **(H–J)**: Major proximal stent edge dissection is seen with the dissection flap extending to media, dissection angle of 180° and 4.3 mm dissection length on three-dimensional (3D) reconstruction. OCT with its better axial resolution enables clearer and more frequent visualization of in-stent tissue prolapse **(K)**.

Stent expansion is adequate if the lesion segment is expanded to a diameter close to or equal to that of a normal artery. It is measured as the minimum stent cross-sectional area either as an absolute value (absolute expansion) or in comparison with a predefined lumen area, which can be the proximal, distal, maximal, or average lumen area (relative expansion). Relative stent expansion is often calculated as the MSA divided by the mean luminal cross-sectional area. Absolute expansion cutoffs appear to be a better predictor of future stent patency than relative expansion (48). Post-PCI MSA has been the most consistent and strongest parameter to predict both restenosis and stent thrombosis (49). Soeda et al. (50) reported that OCT-MSA was an independent predictor of clinical endpoints and target lesion revascularization (TLR) with an MSA cutoff value of 5.0 mm^2^ for DES. Although the DOCTORS trial (16) put the optimal cut-off to predict postprocedural FFR >0.90 in non-left main (LM) arteries at >5.44 mm^2^, Prati et al. in the CLI-OPCI II (12) study showed that MSA <4.5 mm^2^ was associated with MACEs. Modern OCT software (Aptiveu software, OPTIS systems) provides automatic measurement of MSA, stent expansion, and the detection of under-expanded segments. The colored expansion indicator automatically displays red for the under-expanded region and silver for the well-expanded region. UES is diagnosed on OCT when the MSA is <80% of the mean lumen area and/or <4.5 mm^2^ (48) (Figures 3A,B). Stent under-expansion has been established as a major predictor of stent failure (51, 52), and greater absolute stent expansion has been associated with better long-term stent patency, better clinical outcomes, and a lower risk of stent failure (49, 52–54). In addition to diagnosis, OCT also guides the management of UES by providing insights into etiology, such as poorly prepared calcified bed (55) (Figures 3B–D). Under-expansion due to calcium can be managed with ultrahigh-pressure balloon dilation, intravascular lithotripsy (IVL) balloon, or stent ablation with rotational atherectomy.

#### Stent Malapposition

Stent malapposition is defined by a lack of contact between at least one stent strut and the intimal surface below the arterial wall in a segment not above the SB (Figures 3E,G). It may either be acute (detected at the time of the stenting) or late (detected during a subsequent follow-up). It is a common finding during post-PCI optimization, observed in 15% of stents by IVUS imaging (56) and perhaps due to better spatial resolution, at a higher rate of 50% by OCT imaging (13). OCT automatically detects apposed (Figure 3F) and malapposed stent struts and marks them white & red in the rendered stent view respectively. This can also be seen as a red bar in the apposition indicator in L mode (Figure 3E). The impact of acute stent malapposition on stent failure rates (i.e., in-stent restenosis (ISR) and stent thrombosis) has been a matter of controversy because there is no clear link between acute malapposition and stent failure in some prospective studies (57, 58). However, *in vitro* studies have shown that malapposition to be associated with increased thrombogenicity (59) and recent registries have shown that malapposition is a frequent finding in-stent thrombosis (60–62). We recommend the correction of significantly malapposed stent struts with post-dilatation, especially at the proximal stent edge, because this may interfere with rewiring and increase the risk of accidental abluminal rewiring. If the malapposition distance from the endoluminal lining of the strut to the vessel wall is <250 μm, such struts are likely to come into contact with the vessel wall during a follow-up. Therefore, small malapposition may be less relevant (63, 64). In the absence of prospective validation, EAPCI investigators have suggested that acute malapposition of <400 μm with longitudinal extension <1 mm should not be corrected because spontaneous neointimal integration is anticipated (48).

#### Edge Dissection

Deployment of a stent in the coronary artery can sometimes result in unintended tearing of the vessel wall adjacent to the stent struts, resulting in the dissection of the stent edge. Most of these cases occur as a result of landing the stent in unhealthy coronary segments or using oversized stents as compared to the landing zone. Due to its higher resolution, OCT can identify less extensive edge dissections, which are often missed by IVUS and angiography. The ILUMIEN-3 trial reported that the number of dissections detected by OCT was two times that of IVUS. Overall, the incidence of OCT detected edge dissection was as high as 37.8% and most (84%) were not apparent on angiography (65). Although data are limited but not flow-limiting, small dissections have been found to have no impact on the clinical outcomes. Major stent edge dissections (Figures 3H–J) have been defined by their depth (disrupting at least the medial layer), their lateral extension (>60°), and their length (>2 mm) and have shown to be a predictor of poor outcomes (13, 40, 41, 66). Intramural and extramural hematomas detected on OCT usually appear as edge stenosis on angiography and can be misdiagnosed as stent vessel mismatch or spasm.

#### Plaque Prolapse

OCT, with its better axial resolution, enables clearer and more frequent visualization of tissue prolapse (Figure 3K) compared to IVUS (17). The increasing magnitude of prolapse is associated with a higher frequency of TCFAs, plaque rupture, and intracoronary thrombus formation. The identification of a large-volume plaque prolapse immediately after PCI has been associated with post-PCI myocardial injury (67). It has been identified as a predictor of early stent thrombosis and has been related to worse short-term prognoses following PCI (68, 69). Plaque prolapse may be divided into three groups based on severity: smooth protrusion, disrupted fibrous tissue protrusion, and irregular protrusion. Smooth protrusion represents minimal vessel injury, disrupted fibrous tissue protrusion represents mild vessel injury, and irregular protrusion represents a moderate-to-severe vessel injury with a high likelihood of medial disruption and lipid core penetration. Irregular protrusions have been shown to be an independent predictor of 1-year clinical outcomes, primarily driven by TLR (50).

## Oct In Acute Coronary Syndrome

Several of the challenges faced during PCI in a patient with acute coronary syndrome can be overcome with OCT. Around 4–10% of the STEMI patients and >30% of NSTEMI patients present without an identifiable culprit lesion and >10% of patients may have multiple culprit lesions on angiography. OCT not only helps to identify the culprit lesions, especially when the lesions are equivocal on angiography, but also helps infer the underlying mechanisms in ACS and rationalize the decision-making for intervention (70). The most common mechanisms responsible for ACS are plaque rupture and plaque erosion while fewer conditions include thrombosis triggered by calcified nodules, spontaneous dissection, and stent thrombosis. Because of its higher resolution, OCT has a clear advantage over IVUS in identifying these pathological mechanisms of ACS.

### Plaque Rupture

**Figure 4:**
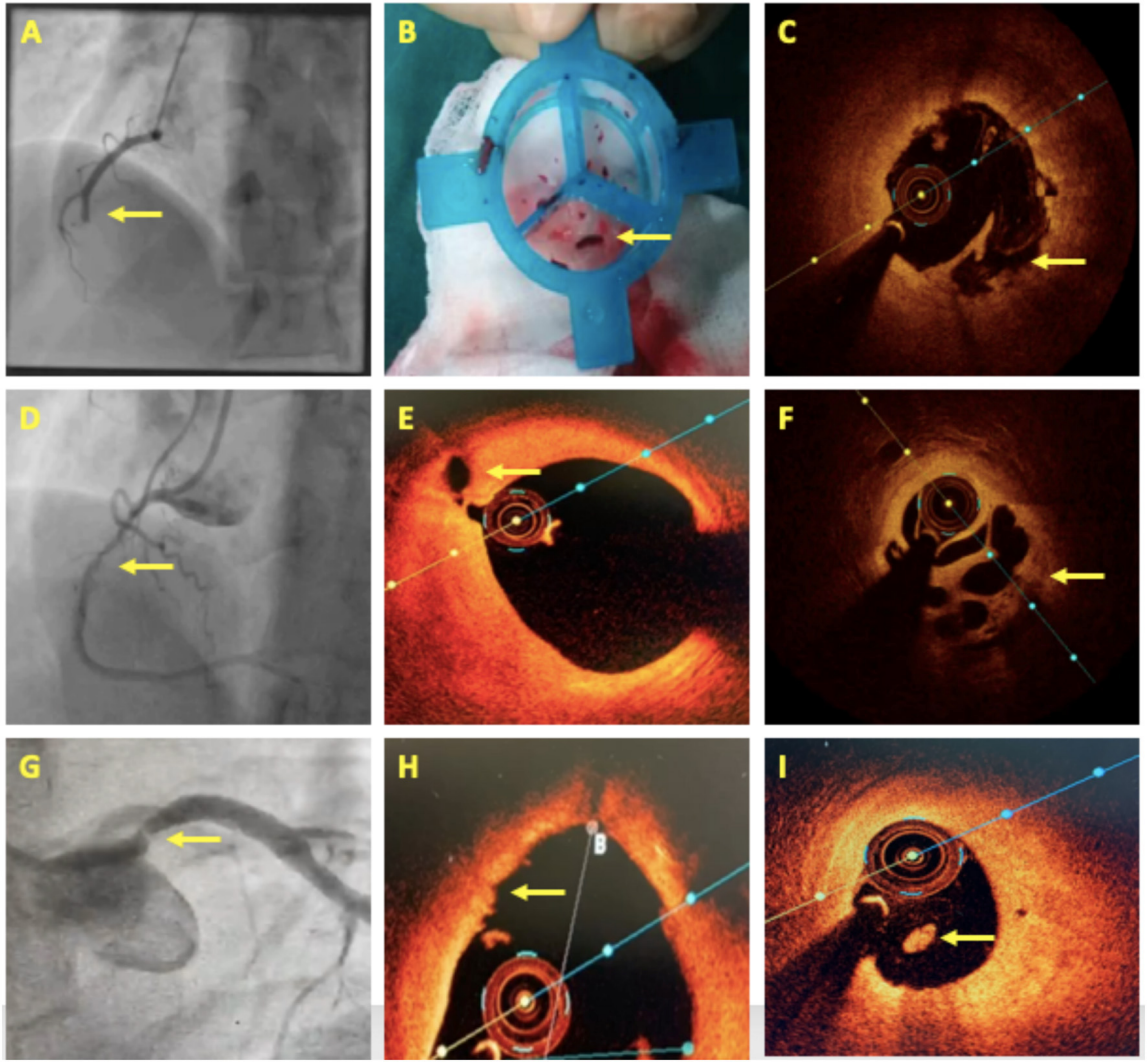
OCT in acute coronary syndrome. **(A–C)** A case of acute inferior wall STEMI with mid dominant right coronary artery (RCA) thrombotic occlusion. Thrombus aspiration followed by OCT revealed plaque rupture. **(D–F)** A 4-day old case of inferior wall STEMI showing Mid-RCA haziness. OCT revealed proximal RCA plaque rupture and mid-RCA recanalized thrombus in a Swiss-cheese pattern. **(G–I)** A case of NSTEMI having a separate origin of LAD and LCx with a significant stenosis in the proximal LAD. OCT showed luminal irregularities, intact thick fibrous cap, and intraluminal white thrombus suggestive of plaque erosion. Areas of interest are highlighted with arrows.

Plaque rupture is the most common cause of ACS, implicated in around 60–70% of the cases. The ruptured plaque is identified by the presence of a discontinuity in the fibrous cap overlying a lipid-rich core and is better seen with OCT (Figures 4C,E). As it ruptures, the contents of the necrotic core come in contact with the bloodstream and trigger the formation of a thrombus that is most often seen adjacent to the ruptured plaque (Figure 4F). Plaque rupture is the more common mechanism of ACS in STEMIs (Figures 4A–F), as opposed to erosion, which is more common in patients with NSTEMI, or calcified nodules that are exclusively observed in NSTEMI (71). Also, fibrin-rich red thrombus (Figure 1H) is frequently found over the ruptured plaque, whereas platelet-rich white thrombus (Figure 1I) is the predominant type of thrombus that forms over erosion and calcified nodules (71).

### Plaque Erosion

Plaque erosion (Figures 4G–I) is seen to be the cause of ACS in 20–30% of cases and tends to occur more in younger patients, especially premenopausal women, current smokers, and patients with the absence of traditional coronary risk factors. These patients generally have a single-vessel disease with reduced lesion severity, larger vessel size, and lesions approaching near bifurcations (70). Pathological studies have defined plaque erosion as a loss of endothelial lining in the absence of “trans-cap” ruptures (31). However, this endothelial loss cannot be visualized directly using OCT at present, and hence, the pathological definition of erosion cannot be simply adapted to OCT. In 2013, Jia et al. (71) defined plaque erosion on OCT as the absence of fibrous cap disruption and further classified it into (1) definite OCT erosion—the presence of attached white thrombus overlying an intact and visible plaque; (2) probable OCT erosion—(a) luminal surface irregularity at the lesion site in the absence of thrombus or (b) the attenuation of underlying plaque by thrombus in the absence of superficial lipid or calcification immediately proximal or distal to the site of thrombus. NSTEMI is the predominant presentation in patients with plaque erosion. Lipid is less frequently detected in erosion than in plaque rupture, although if at all lipid is visualized underneath an erosion, the overlying fibrous cap tends to be thicker, lipid arc tends to be smaller, and lipid length is shorter compared with plaque rupture (71). Plaque rupture is also a highly thrombogenic event and induces massive thrombus burden at the site, causing more luminal obstruction and consequently greater damage. In contrast, plaque erosion seems to result in less thrombus burden, the preservation of vascular structure, and a larger residual lumen (72). Thus, if plaque erosion is treated with aggressive antithrombotic treatment, stent implantation can be avoided, thereby preventing both early and late complications associated with stents. The erosion study showed that the majority (92.5%) of ACS patients with OCT erosion, who were treated with antiplatelet therapy without stenting remained free from MACE after 1 year (73). This study suggests the possibility of individualized therapy for patients with acute coronary syndromes using OCT to establish etiology.

### Calcified Lesions in ACS

Calcified lesions with disruption of the fibrous cap and without lipid core rupture are observed in 7–10% of patients with ACS. Three distinct types of culprit calcified plaques have been described in patients with ACS. (1) Eruptive calcified nodules, which are defined by the extrusion of small calcific nodules into the lumen, (2) superficial calcific sheet, which is the most prevalent type and is characterized by poor baseline TIMI flow and reduced luminal diameter, and (3) calcified protrusion, which is defined by protruding calcific mass without eruptive nodules. All three calcific lesion types are clearly identifiable on OCT and are associated with more periprocedural complications and suboptimal PCI results compared with non-calcified culprit plaques. Eruptive calcified nodules are frequently located in the mid-RCA, where the cyclic hinge movement of the heart may cause weakening of calcified plaques, leading to fractures, whereas superficial calcific sheets are most frequently found in the left anterior descending (LAD) (68.4%) (74).

### Spontaneous Dissection

Spontaneous coronary artery dissection (SCAD) is a rare event leading to an ACS. OCT is extremely useful in its diagnosis as it easily visualizes the double lumen morphology in the vessel wall that is characteristically seen in this condition. We can see the entry tear as well as the circumferential and longitudinal extent of involvement. The compromise of the true coronary lumen and the extent of the false lumen can also be visualized. OCT can also help in wiring through the true lumen for stent implantation. However, performing an OCT in SCAD is not without risks. Because OCT involves the injection of contrast into the coronary lumen, it can result in an extension of the dissection and further compromise the situation. Therefore, dye injection should be performed very carefully and at low flow rates. The recent ESC/Acute Cardiac Care Association (ACCA) position paper on SCAD supports the role of intravascular imaging when angiographic diagnosis is uncertain (75).

## Oct For Stent Failure

Optical coherence tomography is the investigation of choice in the assessment of stent failure. ISR and stent thrombosis, along with scaffold thrombosis, are the main causes of stent failure. OCT images provide insights into the mechanism of stent failure and thus the selection of an appropriate management strategy.

### In-stent Restenosis

**Figure 5:**
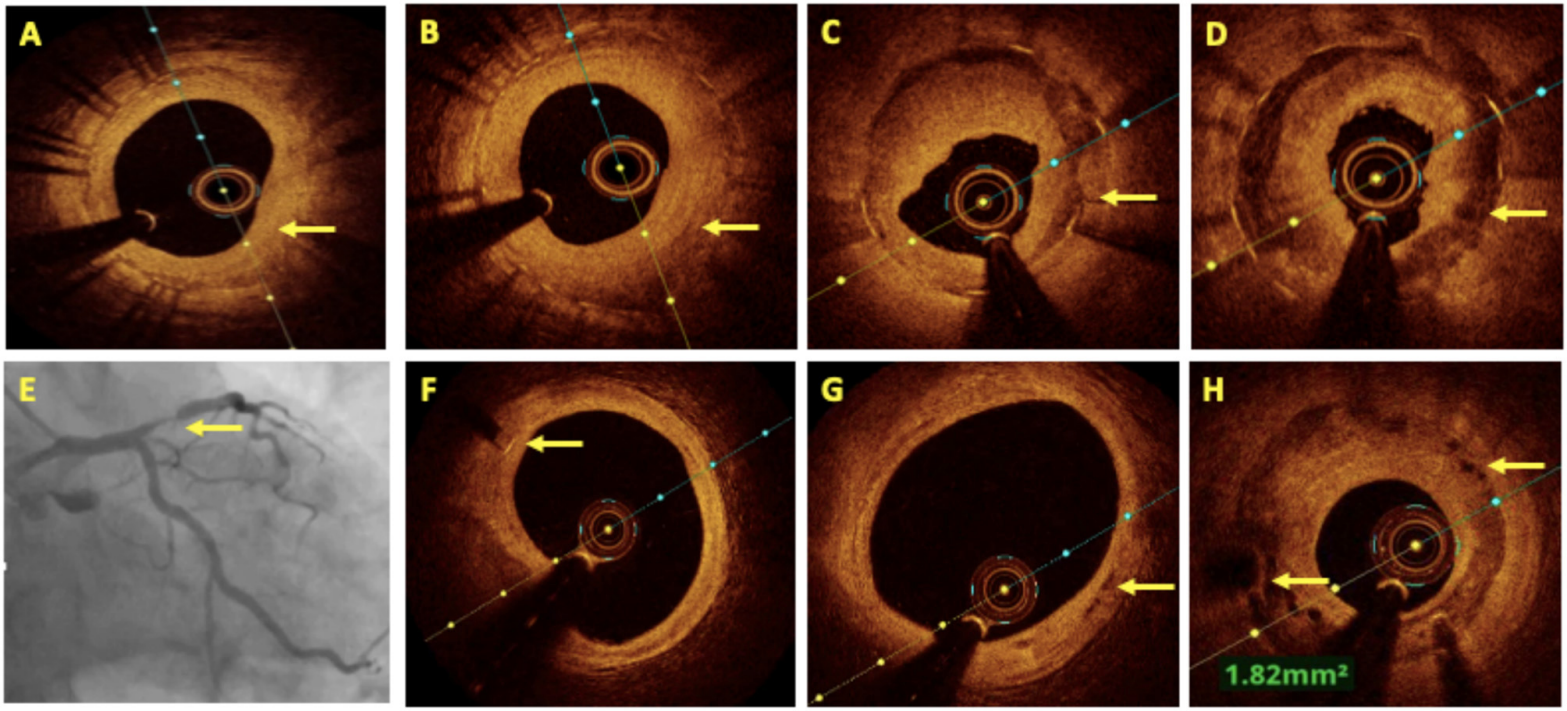
OCT in in-stent restenosis (ISR). **(A)** Homogenous neointimal hyperplasia (NIH) in bare metal stent (BMS) restenosis. **(B)** Heterogenous NIH in a drug-eluting stent (DES) ISR. **(C,D)** Showed neoatherosclerosis as a cause of ISR in a well-expanded stent with well-demarcated calcium and fibrotic ingrowth. **(E–H)** Coronary angiography showing proximal LAD bioresorbable vascular scaffolds (BVS) ISR after 2 years of implantation. **(F)** The OCT image shows a distal marker of BVS, **(G)** almost completely absorbed BVS struts in the middle and distal part of the stent, and **(H)** BVS ISR at the proximal segment with peri-strut low intensity areas.

Arterial healing after stenting is best studied by OCT. ISR is the most common cause of stent failure, and although its incidence has reduced drastically compared to the bare metal stent (BMS) era, ISR has been described in the latest generation stents and remains a challenge. OCT can reveal the etiology of an ISR such as under-expansion, malapposition, or strut fracture. It can also characterize ISR as NIH or neoatherosclerosis. NIH associated with BMS is homogenous (Figure 5A), whereas DES-related NIH is more heterogenous (Figure 5B) (76). Neoatherosclerotic hyperplasia as a cause of ISR (Figures 5C,D) is characterized by lipid and/or calcium deposition along with a necrotic core and even infiltration by macrophages. Indeed, in some cases, it is difficult to distinguish a DES-related ISR from TCFA lesions in native plaques (77). The treatment of choice for ISR remains the implantation of DES, particularly everolimus-eluting DES, which has consistently been shown to be superior to drug-eluting balloon (DEB) in terms of short- and long-term outcomes (78, 79). While DEB is associated with higher clinical and angiographic restenosis rates, probably due to its inability to achieve maximum acute luminal gain (80), the addition of another metallic layer associated with DES implantation may also be undesirable in these patients. Recently, available BVS address both concerns. BVS provides a temporary vascular scaffolding to maximize the acute luminal gain without adding an additional permanent layer of metal. OCT is preferred over IVUS for BVS implantation and to study tissue coverage in a follow-up (Figures 5E–G) (81, 82). A 3-year follow-up intracoronary OCT study demonstrated complete tissue coverage of the scaffold (83). Although data regarding the efficacy of BVS in the treatment of ISR is conflicting, recent evidence may point toward outcomes trending closer to DES (84). Another study with a 1-year follow-up after using BVS for ISR treatment showed a non-significant higher incidence of device-related cardiovascular events compared to EES (10.1 vs. 5.2%, *p* = 0.27) (85). BVS indeed looks to be a viable option in the treatment of ISR.

In the treatment of native coronary artery stenosis, complete scaffold resorption may theoretically lead to improved clinical outcomes that are very late compared to metallic-frame DES. However, although 1-year follow-up results showed non-inferiority to EES (86), more studies have demonstrated an accumulating risk of BVS-related thrombosis and MI between 1 and 3 years (87–89). Recently, a 5-year follow-up of the ABSORB III trial showed that the period of excess risk for BVS ended at 3 years, coinciding with the time of complete scaffold resorption (90). ISR after BVS implantation (Figure 5H) is also a known entity, and studies have suggested that its 3-year incidence becomes comparable to that associated with DES implantation when using optimal implantation techniques with intracoronary imaging (91).

### Stent Thrombosis

One of the most serious complications of stent implantation is stent thrombosis, which can adversely affect life expectancy and lead to myocardial infarction. Although the incidence of stent thrombosis is low in the present era, a large number of patients undergoing DES implantation make it a significant problem. OCT in the setting of acute/subacute stent thrombosis has been shown to be feasible. The most common findings in these cases are the presence of uncovered struts and malapposed struts along with under-expansion of the stented coronary segment. These are identified as the key morphological features of stent thrombosis by OCT (60). The presence of a non-streamlined blood flow along malapposed stent struts has been shown to be one of the factors related to acute stent thrombogenicity (92). Additionally, residual stenosis within the stented segment or small MLA is also an independent and well-recognized predictor of stent thrombosis (50). In the setting of late/very late stent thrombosis, within the 1st year of intervention, uncovered/malapposed stent struts, under-expansion, and severe restenosis predominate the OCT findings, and ISR with neo-atherosclerosis predominates beyond 1 year. These findings definitively suggest that improved recognition and correction of suboptimal stent deployment is likely to significantly impact stent thrombosis rates.

## Oct In Bifurcation Lesions

**Figure 6:**
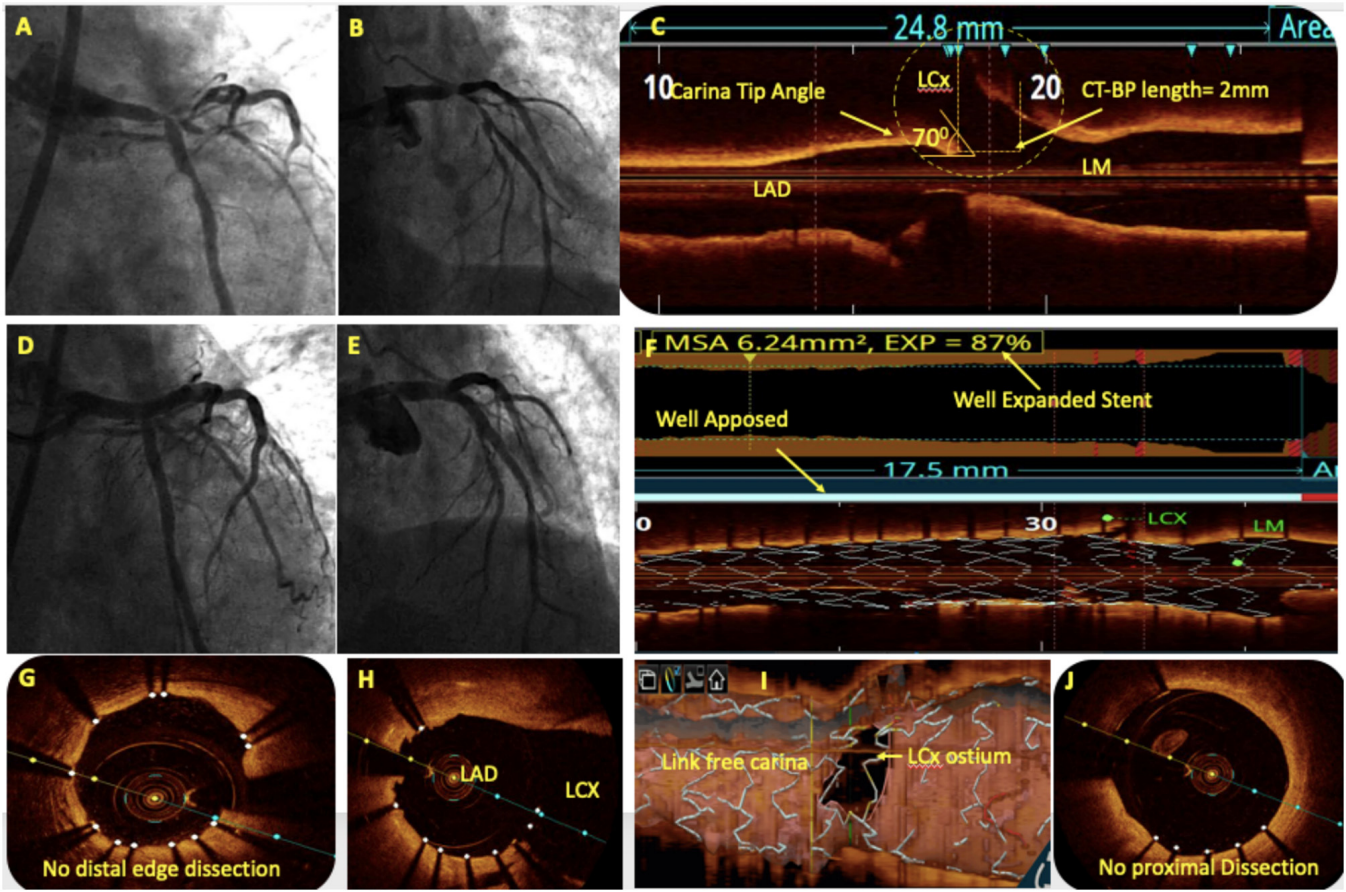
OCT in bifurcation angioplasty. **(A,B)** Coronary angiography showed a significant left main (LM) bifurcation lesion (Medina 1.1.1). **(C)** Pre-PCI OCT showed the carina tip (CT) angle of 70° and CT to bifurcation point (CT-BP length) of 2 mm suggestive of bifurcation lesion suitable for provisional stenting without the risk of side branch (SB) compromise. **(D)** An OCT-guided LM-LAD cross-over stenting was done followed by the proximal optimization technique (POT) with balloons of appropriate size. **(E)** Post-PCI angiography showed TIMI III flow in left coronary system without LCx compromise. Post-PCI OCT showed well-apposed stent with 87% expansion **(F)**, without proximal or distal edge dissection **(G,J)** and minimal stent struts across LCx ostium **(H)**. 3D reconstruction showed minimal inappropriate stent apposition across LCx ostium with link-free carina **(I)**.

**Figure 7:**
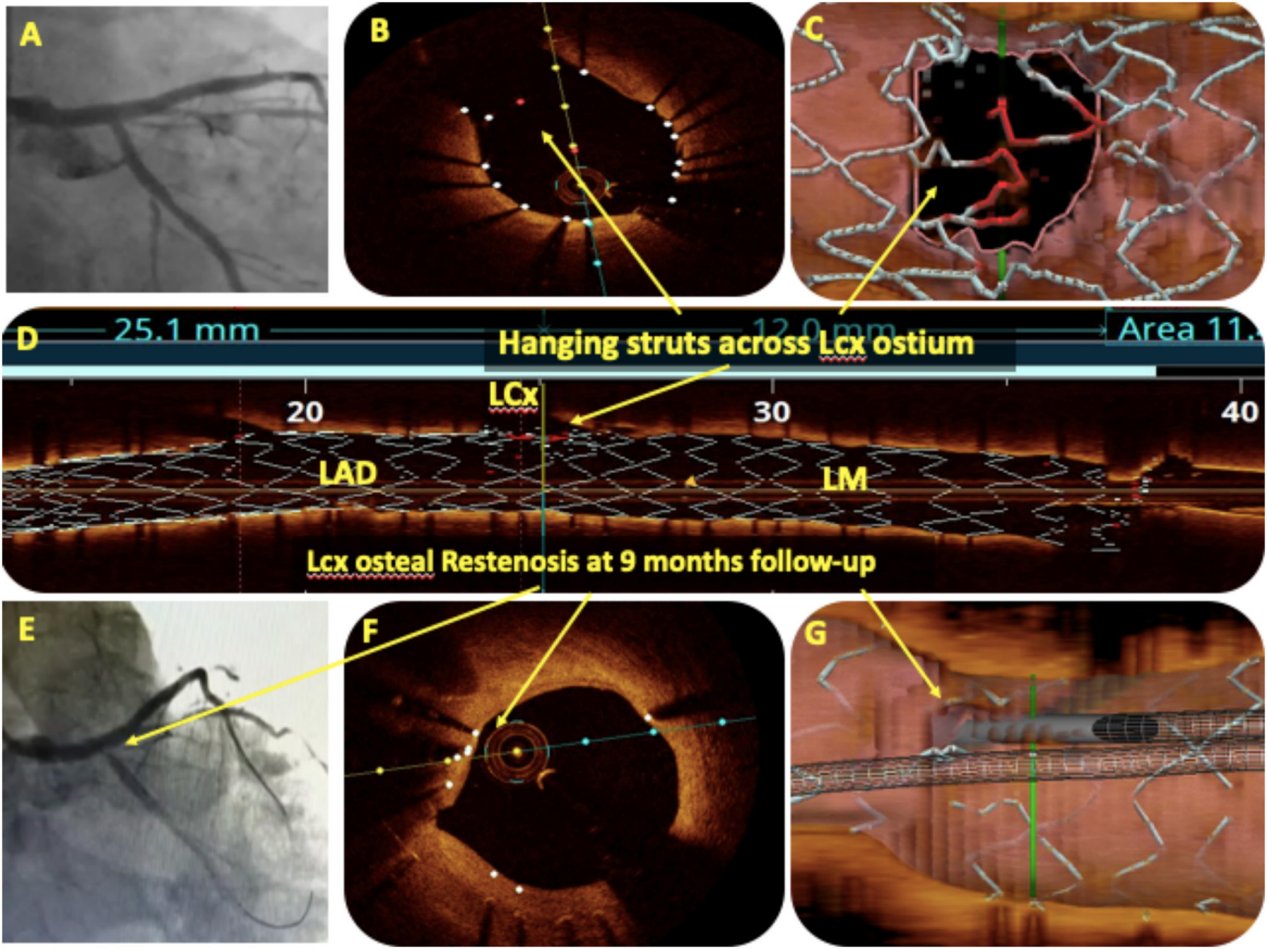
OCT in bifurcation angioplasty. **(A)** Showed the final angiogram picture after LM bifurcation angioplasty with two stents using the DK crush technique. The angiographic result for LCx ostium was satisfactory but OCT pullback of the main branch (MB) after final kissing balloon inflation (KBI) showed hanging stent struts (red) across LCx ostium on the cross-sectional view **(B)**, on 3D reconstruction **(C)**, and on the rendered stent view **(D)**. The same patient presented with crescendo angina after 9 months, and angiography showed LCx ostial ISR **(E)**. OCT pullback of MB showed complete endothelization of hanging stent struts across LCx as a cause of ostial ISR **(F,G)**.

**Figure 8:**
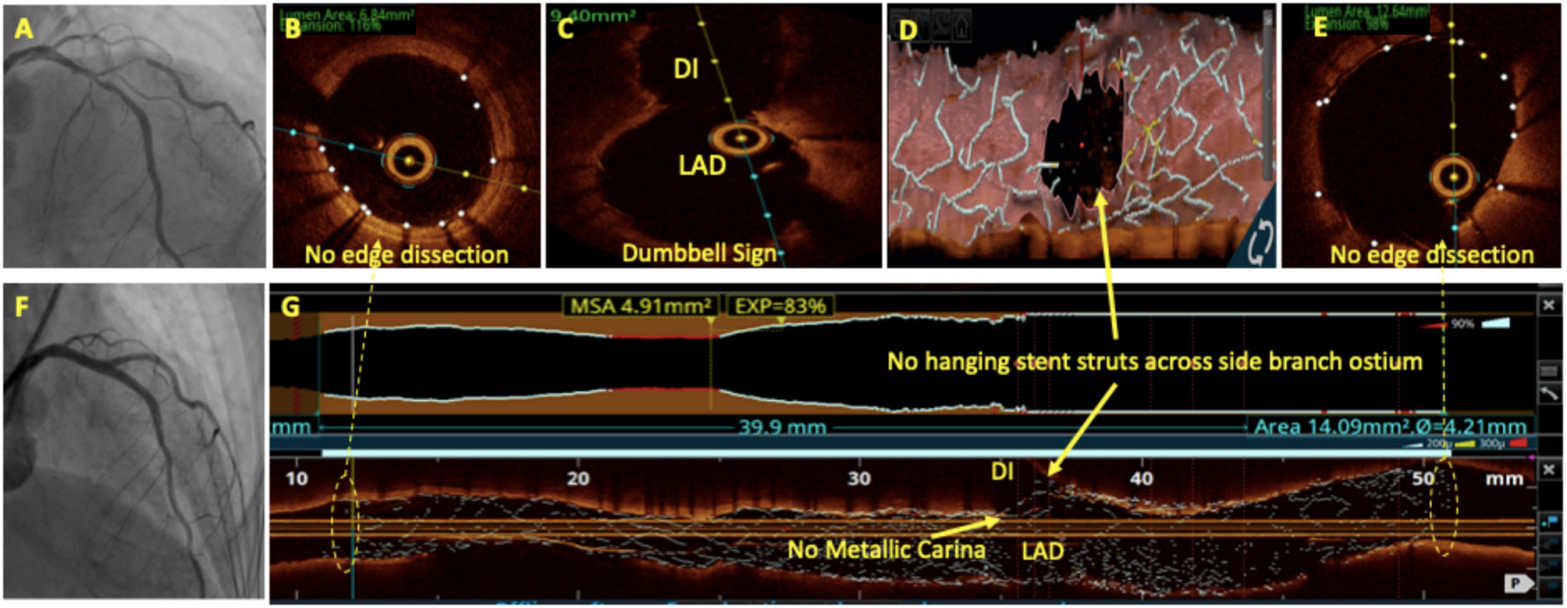
OCT in bifurcation angioplasty. Coronary angiography showed a significant LAD/D1 stenosis **(A)** with Medina class (1, 1, 1). Patient underwent bifurcation angioplasty with two stents using the DK crush technique **(F)**. OCT run of LAD after final KBI showed no edge dissection **(B,E)** and dumbbell sign **(C)**. No hanging stent struts seen across D1 ostium on 3D reconstruction **(D)** and on L mode **(G)**. No neo-metallic carina seen at LAD/D1 bifurcation on the rendered stent view **(G)**.

Bifurcation lesions are usually complex and comprise 15–20% of all PCI procedures (93) and pose unique procedural and outcome challenges. For provisional bifurcation angioplasty, in addition to routine pre-PCI OCT assessment, the three important parameters that need to be recorded are plaque distribution in relation to SB ostium, carina tip (CT) angle, and CT to bifurcation point (BP) length (Figure 6). CT angle <50° and CT-BP length <1.7 mm have been found to be independent predictors of SB complications in the provisional strategy (94). Predominantly calcified plaque opposite to SB ostia is a predictor of SB compromise after MB stenting due to carinal shift. MB distal reference diameter guides the stent size while the proximal reference diameter guides the size of the proximal optimization technique (POT) balloon. Post-PCI MB OCT pullback is done to assess stent expansion, apposition, and edge dissection. 3D reconstruction at the bifurcation clearly quantifies the stent struts across SB ostium, identifies whether carina is link-free or not, and helps in distal guide wire recrossing. A few studies have shown that link-free carina with distal SB wiring resulted in minimum inappropriate stent apposition or neocarina across the SB ostium after kissing balloon inflation (KBI) although it did not translate into improved clinical outcomes (95). For upfront two stent strategy, pre-PCI OCT assessment is done in both SB and MB. The distal reference diameter of both SB and MB is used to decide the size of both stents and the proximal reference diameter of MB is used for sizing of the POT balloon. Post-PCI OCT pullbacks of both MB and SB are taken to assess stent expansion, apposition, edge dissection, and plaque protrusion. MB OCT pullback after final KBI should be used to analyze the SB ostium. Hanging stent struts across SB ostium can serve as nidus for SB restenosis due to endothelization (Figure 7). Hence, it should be ensured that SB ostium is free from hanging stent struts in the 3D reconstruction, with minimal neo-metallic carinal length in the L mode and the dumbbell sign adjacent to the native carina in the cross-sectional view (Figure 8).

Side branch ostial dimensions can also be assessed by an MB pullback, but often, due to the non-coaxial position of the OCT catheter relative to the SB or to extreme angulation between the SB and the MB, the error in the assessment of the SB ostium area is particularly high and should be avoided. Recently, the assessment of SB ostium using the “cut plane analysis” *via* a dedicated “QAngioOCT” software has shown to significantly reduce the error in the assessment of SB ostium and provides highly reliable and reproducible measurements (96).

### OCT in LM Bifurcation Lesions

LM lesions display specific features (diameter discrepancies between MB and SB, tapered anatomy, plaque eccentricity, and increased likelihood of calcifications) that are difficult to analyze correctly with angiography alone. Guidelines recommend IVUS for LM angioplasty, principally because of the theoretical limitations of OCT in imaging the LM body due to its bigger size and the LM ostia due to poor blood clearance. Recently, many trials have investigated OCT-guided mid and distal LM PCI with the results comparable, if not superior, to IVUS (97, 98) and also demonstrated that 81% of OCT image frames in LM PCI were analyzable and that most of the non-analyzable, artifact frames were in the proximal LM (97).

The ROCK-1 (99) retrospective study demonstrated that a substantial number of acute strut malapposition and stent under-expansion in bifurcation stenting were detected under OCT guidance. Compared with standard angiographic guidance alone, late lumen loss in a 6-month follow-up tended to be lower in the OCT group (0.12 ± 0.41 vs. 0.26 ± 0.52 mm, *p* = 0.10), and was significantly reduced in the distal portion of the main vessel (0.03 ± 0.45 vs. 0.24 ± 0.53 mm, *p* = 0.025). In the 70 patients included in the final analysis of the LEMON study (100) which aimed to analyze the feasibility, safety, and impact of OCT-guided LM PCI, the primary endpoint of procedural success was achieved in 86% of subjects. Adequate stent expansion was observed in 86%, significant edge dissection in 30%, and residual significant strut malapposition in 24% of the cases. OCT guidance modified operators' strategy in one out of four despite experienced operators and acceptable angiographic results. Additionally, 1-year survival free from major clinical adverse events was 98.6%. Therefore, we recommend OCT for distal LM bifurcation angioplasty performed either provisionally or with a two-stent strategy upfront as it can image most sizes of LM encountered by the interventionalist and has the distinct advantage of post-PCI SB ostial optimization compared with IVUS.

**Figure 9:**
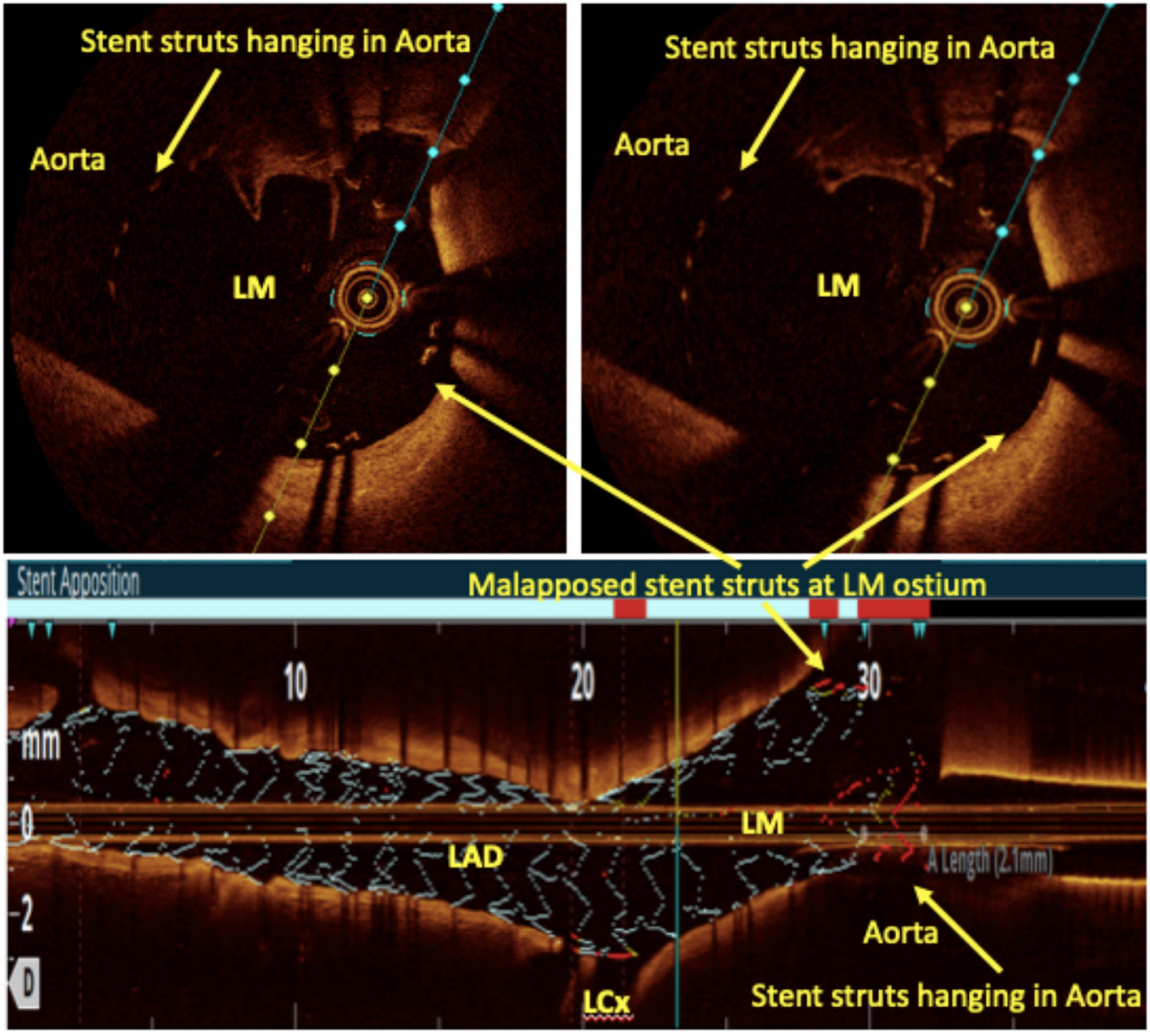
Left main ostial imaging with OCT. OCT pullback from LAD to LM after provisional LM ostial to LAD crossover angioplasty with one wire in the aorta. Hanging stent struts in the aorta (suggestive of LM ostial coverage) can be clearly seen along with some malapposed struts both in the cross-sectional and rendered stent view.

An aorto-ostial lesion, which has been a significant limitation of OCT, has recently been observed with the help of the soft polymer tip of the guide-extension catheter named “Telescope” (Medtronic Cardiovascular, Santa Rosa, CA, USA). This catheter has a helical coil constitution with a relatively wide gap that enables the penetration of infrared (IR) light through the soft polymer. It is certainly a step forward into the uncharted territory of OCT-guided ostial LM stenting. LM ostia can also be imaged by placing a guidewire in the aorta and aligning the guide catheter to the coronary ostia (Figure 9).

## Oct For Coronary Artery Aneurysm

In comparison to IVUS, OCT has a limited role in imaging large coronary artery aneurysms (CAAs), but it can provide valuable inputs such as differentiation between true aneurysms or pseudoaneurysms and the state of adjacent coronaries in small CAAs. The introduction of DES has led to an increasing number of reports of stent-related aneurysms that can sometimes be fatal (101). This may be partly explained by the antiproliferative action of DES, which may delay neointimal healing after vessel wall injury, thus predisposing the vessel to aneurysm formation. CAAs can be managed with covered stents, and an “eclipse sign” has been described post-stenting in a CAA due to the eclipse-like appearance of the covered stent OCT (102).

## Contrast Sparing Oct

**Figure 10:**
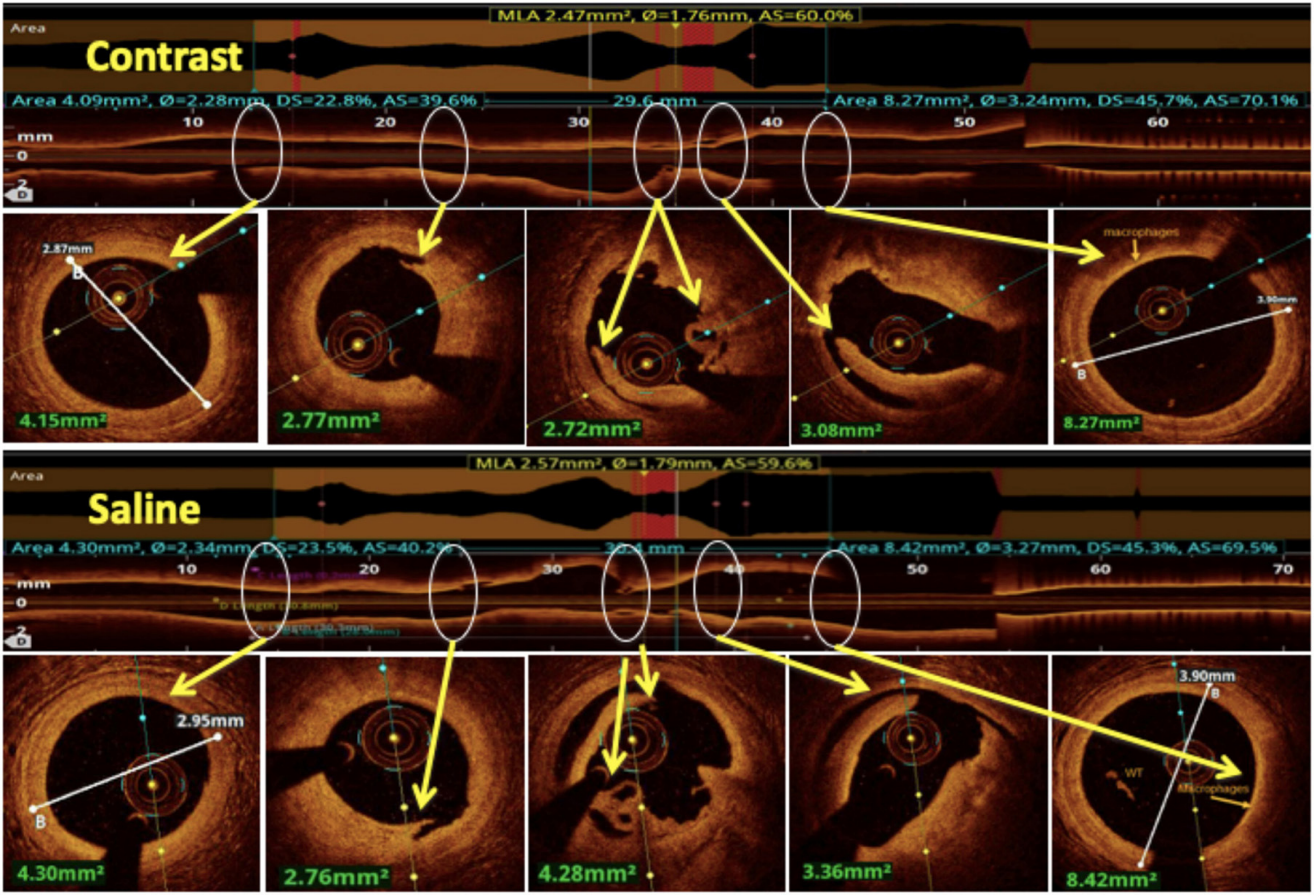
Comparison between saline and contrast OCT. The upper panel shows pre-PCI run of RCA using contrast as a flushing media and in the lower panel the same vessel is imaged using saline as a flushing media for OCT and compared for image quality. All the lesion morphologies (including plaque rupture, dissections, recanalized thrombus, and macrophages) seen in contrast OCT are clearly seen with saline OCT (marked by yellow arrows at the same level).

In patients who have baseline renal dysfunction or are at a higher pre-procedural risk of developing contrast-induced nephropathy, novel contrast sparing techniques have been developed to permit the use of OCT in coronary revascularization. Low-molecular-weight dextran (LMWD) has been used as a substitute for contrast to clear the coronary blood field, and the same image quality and quantitative assessment has been achieved (103, 104). The use of OCT with LMWD has also been studied in patients with CKD, in whom the use of LMWD did not adversely affect renal function and achieved similar short- and long-term clinical outcomes compared to IVUS-guided PCI (105). However, its use is limited by the availability and risk of anaphylactoid reactions (106). Another medium that has been studied as an alternative to contrast is heparinized saline which has recently been demonstrated to be feasible and safe with acceptable image quality (107). One study showed that coronary dimensions measured using saline OCT were comparable to those obtained with contrast and hence saline OCT may be used as a contrast alternative for coronary OCT during PCI optimization (108) (Figure 10). Further studies in this field are needed to provide firm evidence on the feasibility of saline as a flushing media.

## Future Of Oct

### Artificial Intelligence

It has emerged largely to positively enable the way we work, especially in the field of imaging, and a novel AI framework has been developed for automatic plaque characterization in OCT. Ultreon 1.0 software automatically detects the severity of calcium in lesions and measures the vessel diameter. Both internal and external validation have also been obtained. Chu et al. found that AI-enabled OCT had excellent consistency in quantifying plaque burden compared to manual measurements (109).

### Development of a Hybrid OCT–IVUS Catheter

OCT and IVUS as individual guiding tools have certain limitations. However, combining the strengths of the two imaging techniques into one may create the most comprehensive intracoronary imaging guidance available. The initial prototypes were in development since 2011 and, after many animal and cadaveric studies, a hybrid IVUS–OCT catheter was first put into clinical application by Sheth et al. (110) in 2018, where the IVUS and OCT beams were aligned with each other, allowing for inherent co-registration and immediate simultaneous image acquisition and review. Lipid-laden plaques, BPs, and deeper tissues were more clearly identified with IVUS, whereas calcifications, stent struts, and fine dissections were more clearly identified with OCT imaging. Currently, the system does not allow stand-alone OCT acquisition. This catheter powered by Novasight system technology (Conavi Medical, Inc. Toronto, Canada) demonstrated the synergistic ability of the two modalities to characterize coronary atherosclerosis and produce high-quality co-registered images with clinically acceptable specifications with respect to the dimensions, speed, and resolution.

Another hybrid IVUS–OCT catheter system with dual sensors in a sequential arrangement consisting of an IVUS transducer and an optical lens has been developed by TERUMO (Tokyo, Japan) by merging IVUS and optical frequency domain imaging (OFDI) probes. In this hybrid catheter, IVUS or OCT can be used separately according to the circumstances during the procedure. For example, in the case of LM disease, the IVUS function can be used to assess the severity and guide treatment, whereas OCT can be utilized to assess wire recrossing, final results, and detect and treat underexpansion/malapposition and/or carina shift at the bifurcation.

## Conclusion

Intracoronary OCT has been shown to be clearly more advantageous than angiographic guidance and, although its use results in clinical outcomes similar to IVUS guidance, the in-depth data and better visualization of the lesion morphology provided by OCT contribute to better intraprocedural decision-making capabilities. OCT is more sensitive in detecting vulnerable plaques, quantifying calcified lesions, guiding SB recrossing in bifurcation stenting, intrastent tissue protrusion, incomplete stent apposition, stent edge dissections, and intrastent thrombus when compared with IVUS. Further evidence on the clinical and long-term benefits of OCT guidance is anticipated from the ongoing large-scale ILUMIEN IV and OCTOBER trials. OCT overcomes its traditional pitfalls with the advent of saline-based OCT and ostial LM visualization with the advent of the “telescope” catheter, albeit in developmental stages. Improved clinical outcomes and awareness among interventionalists should increase the adoption of this imaging modality in clinical practice.

## Author Contributions

All authors listed have made a substantial, direct, and intellectual contribution to the work and approved it for publication.

## Conflict of Interest

The authors declare that the research was conducted in the absence of any commercial or financial relationships that could be construed as a potential conflict of interest.

## Publisher's Note

All claims expressed in this article are solely those of the authors and do not necessarily represent those of their affiliated organizations, or those of the publisher, the editors and the reviewers. Any product that may be evaluated in this article, or claim that may be made by its manufacturer, is not guaranteed or endorsed by the publisher.

## Supplementary Material

Supplementary Video 1 Optical coherence tomography (OCT) pullback of left anterior descending artery with real-time angiography co-registration (ACR). OCT frames corresponds to the location of a marker moving in the left anterior descending (LAD) artery.

## References

[1] Topol EJ Nissen SE. Our preoccupation with coronary luminology. The dissociation between clinical and angiographic findings in ischemic heart disease. Circulation. (1995) 92:2333–42. 10.1161/01.CIR.92.8.2333 7554219

[2] Zir LM Miller SW Dinsmore RE Gilbert JP Harthorne JW. Interobserver variability in coronary angiography. Circulation. (1976) 53:627–32. 10.1161/01.CIR.53.4.627 1253383

[3] White CW Wright CB Doty DB Hiratza LF Eastham CL Harrison DG. Does visual interpretation of the coronary arteriogram predict the physiologic importance of a coronary stenosis? N Engl J Med. (1984) 310:819–24. 10.1056/NEJM198403293101304 6700670

[4] Mintz GS. Clinical utility of intravascular imaging and physiology in coronary artery disease. J Am Coll Cardiol. (2014) 64:207–22. 10.1016/j.jacc.2014.01.015 24530669

[5] Neumann F-J Sousa-Uva M Ahlsson A Alfonso F Banning AP Benedetto U. 2018 ESC/EACTS guidelines on myocardial revascularization. Euro Heart J. (2019) 40:87–165. 10.1093/eurheartj/ehy855 30615155

[6] Huang D Swanson EA Lin CP Schuman JS Stinson WG Chang W. Optical coherence tomography. Science. (1991) 254:1178–81. 10.1126/science.1957169 1957169 PMC4638169

[7] Jang I-K Bouma BE Kang D-H Park S-J Park S-W Seung K-B. Visualization of coronary atherosclerotic plaques in patients using optical coherence tomography: comparison with intravascular ultrasound. J Am Coll Cardiol. (2002) 39:604–9. 10.1016/S0735-1097(01)01799-5 11849858

[8] Prati F Cera M Ramazzotti V Imola F Giudice R Albertucci M. Safety and feasibility of a new non-occlusive technique for facilitated intracoronary optical coherence tomography (OCT) acquisition in various clinical and anatomical scenarios. EuroIntervention. (2007) 3:365–70. 10.4244/EIJV3I3A66 19737719

[9] Yoon JH Di Vito L Moses JW Fearon WF Yeung AC Zhang S. Feasibility and safety of the second-generation, frequency domain optical coherence tomography (FD-OCT): a multicenter study. J Invasive Cardiol. (2012) 24:206–9. 22562913

[10] Imola F Mallus MT Ramazzotti V Manzoli A Pappalardo A Di Giorgio A. Safety and feasibility of frequency domain optical coherence tomography to guide decision making in percutaneous coronary intervention. EuroIntervention. (2010) 6:575–81. 10.4244/EIJV6I5A97 21044910

[11] van der Sijde JN Karanasos A van Ditzhuijzen NS Okamura T van Geuns R-J Valgimigli M. Safety of optical coherence tomography in daily practice: a comparison with intravascular ultrasound. Euro Heart J Cardiovasc Imaging. (2017) 18:467–74. 10.1093/ehjci/jew037 26992420

[12] Prati F Di Vito L Biondi-Zoccai G Occhipinti M La Manna A Tamburino C. Angiography alone versus angiography plus optical coherence tomography to guide decision-making during percutaneous coronary intervention: the centro per la lotta contro l'infarto-optimisation of percutaneous coronary intervention (CLI-OPCI) study. EuroIntervention. (2012) 8:823–9. 10.4244/EIJV8I7A125 23034247

[13] Prati F Romagnoli E Burzotta F Limbruno U Gatto L La Manna A. Clinical impact of OCT findings during PCI: the CLI-OPCI II study. JACC Cardiovasc Imaging. (2015) 8:1297–305. 10.1016/j.jcmg.2015.08.013 26563859

[14] Antonsen L Thayssen P Maehara A Hansen HS Junker A Veien KT. Optical coherence tomography guided percutaneous coronary intervention with nobori stent implantation in patients with non-ST-segment-elevation myocardial infarction (OCTACS) trial: difference in strut coverage and dynamic malapposition patterns at 6 mon. Circul Cardiovasc Interv. (2015) 8:e002446. 10.1161/CIRCINTERVENTIONS.114.002446 26253735

[15] Wijns W Shite J Jones MR Lee SWL Price MJ Fabbiocchi F. Optical coherence tomography imaging during percutaneous coronary intervention impacts physician decision-making: ILUMIEN I study. Eur Heart J. (2015) 36:3346–55. 10.1093/eurheartj/ehv367 26242713 PMC4677272

[16] Meneveau N Souteyrand G Motreff P Caussin C Amabile N Ohlmann P. Optical coherence tomography to optimize results of percutaneous coronary intervention in patients with non–ST-elevation acute coronary syndrome. Circulation. (2016) 134:906–17. 10.1161/CIRCULATIONAHA.116.024393 27573032

[17] Ali ZA Maehara A Généreux P Shlofmitz RA Fabbiocchi F Nazif TM. Optical coherence tomography compared with intravascular ultrasound and with angiography to guide coronary stent implantation (ILUMIEN III: OPTIMIZE PCI): a randomised controlled trial. Lancet. (2016) 388:2618–28. 10.1016/S0140-6736(16)31922-5 27806900

[18] Jones DA Rathod KS Koganti S Hamshere S Astroulakis Z Lim P. Angiography alone versus angiography plus optical coherence tomography to guide percutaneous coronary intervention: outcomes from the pan-london PCI cohort. JACC Cardiovasc Interv. (2018) 11:1313–21. 10.1016/j.jcin.2018.01.274 30025725

[19] Chamié D Costa JRJ Damiani LP Siqueira D Braga S Costa R. Optical coherence tomography versus intravascular ultrasound and angiography to guide percutaneous coronary interventions: the iSIGHT randomized trial. Circul Cardiovasc Interv. (2021) 14:e009452. 10.1161/CIRCINTERVENTIONS.120.009452 33685212

[20] Hebsgaard L Nielsen TM Tu S Krusell LR Maeng M Veien KT. Co-registration of optical coherence tomography and X-ray angiography in percutaneous coronary intervention. The does optical coherence tomography optimize revascularization (DOCTOR) fusion study. Int J Cardiol. (2015) 182:272–8. 10.1016/j.ijcard.2014.12.088 25585362

[21] Habara M Nasu K Terashima M Kaneda H Yokota D Ko E. Impact of frequency-domain optical coherence tomography guidance for optimal coronary stent implantation in comparison with intravascular ultrasound guidance. Circul Cardiovasc Interv. (2012) 5:193–201. 10.1161/CIRCINTERVENTIONS.111.965111 22456026

[22] Kubo T Akasaka T Shite J Suzuki T Uemura S Yu B. OCT compared with IVUS in a coronary lesion assessment: the OPUS-CLASS study. JACC Cardiovasc Imaging. (2013) 6:1095–1104. 10.1016/j.jcmg.2013.04.014 24011777

[23] Maehara A Ben-Yehuda O Ali Z Wijns W Bezerra HG Shite J. Comparison of stent expansion guided by optical coherence tomography versus intravascular ultrasound: the ILUMIEN II study (observational study of optical coherence tomography [OCT] in patients undergoing fractional flow reserve [FFR] and percutaneous Co. JACC Cardiovasc Interv. (2015) 8:1704–14. 10.1016/j.jcin.2015.07.024 26585621

[24] Kubo T Shinke T Okamura T Hibi K Nakazawa G Morino Y. Optical frequency domain imaging vs. intravascular ultrasound in percutaneous coronary intervention (OPINION trial): one-year angiographic and clinical results. Eur Heart J. (2017) 38:3139–47. 10.1093/eurheartj/ehx351 29121226 PMC5837511

[25] Muramatsu T Ozaki Y Nanasato M Ishikawa M Nagasaka R Ohota M. Comparison between optical frequency domain imaging and intravascular ultrasound for percutaneous coronary intervention guidance in biolimus A9-eluting stent implantation. Circul Cardiovasc Interv. (2020) 13:e009314. 10.1161/CIRCINTERVENTIONS.120.009314 33106049 PMC7665240

[26] Chhikara S Datta R Shrivastava A Gupta A. Stuck OCT catheter: an unanticipated complication. J Invasive Cardiol. (2022) 34:E149–50. 35100558 10.25270/jic/21.00273

[27] Tearney GJ Regar E Akasaka T Adriaenssens T Barlis P Bezerra HG. Consensus standards for acquisition, measurement, and reporting of intravascular optical coherence tomography studies: a report from the international working group for intravascular optical coherence tomography standardization and validation. J Am Coll Cardiol. (2012) 59:1058–72. 22421299 10.1016/j.jacc.2011.09.079

[28] Brezinski ME Tearney GJ Bouma BE Izatt JA Hee MR Swanson EA. Optical coherence tomography for optical biopsy: properties and demonstration of vascular pathology. Circulation. (1996) 93:1206–13. 10.1161/01.CIR.93.6.1206 8653843

[29] Davies MJ. Detecting vulnerable coronary plaques. Lancet. (1996) 347:1422–3. 10.1016/S0140-6736(96)91677-3 8676621

[30] Falk E. Plaque rupture with severe pre-existing stenosis precipitating coronary thrombosis. Characteristics of coronary atherosclerotic plaques underlying fatal occlusive thrombi. Heart. (1983) 50:127–34. 10.1136/hrt.50.2.127 6882602 PMC481384

[31] Virmani R Kolodgie FD Burke AP Farb A Schwartz SM. Lessons from sudden coronary death: a comprehensive morphological classification scheme for atherosclerotic lesions. Arterioscler Thromb Vasc Biol. (2000) 20:1262–75. 10.1161/01.ATV.20.5.1262 10807742

[32] Prati F Romagnoli E Gatto L La Manna A Burzotta F Ozaki Y. Relationship between coronary plaque morphology of the left anterior descending artery and 12 months clinical outcome: the CLIMA study. Eur Heart J. (2020) 41:383–91. 10.1093/eurheartj/ehz520 31504405

[33] Kedhi E Berta B Roleder T Hermanides RS Fabris E IJsselmuiden AJJ. Thin-cap fibroatheroma predicts clinical events in diabetic patients with normal fractional flow reserve: the COMBINE OCT–FFR trial. Eur Heart J. (2021) 42:4671–9. 10.1093/eurheartj/ehab433 34345911

[34] Kedhi E Berta B Roleder T Hermanides RS Fabris E IJsselmuiden AJJ. Percutaneous coronary intervention for vulnerable coronary atherosclerotic plaque. J Am Coll Cardiol. (2020) 76:2289–301. 10.1016/j.jacc.2020.09.547 33069847

[35] Erlinge D Maehara A Ben-Yehuda O Bøtker HE Maeng M Kjøller-Hansen L. Identification of vulnerable plaques and patients by intracoronary near-infrared spectroscopy and ultrasound (PROSPECT II): a prospective natural history study. Lancet. (2021) 397:985–995. 33714389 10.1016/S0140-6736(21)00249-X

[36] Fujino A Mintz GS Matsumura M Lee T Kim S-Y Hoshino M. A new optical coherence tomography-based calcium scoring system to predict stent underexpansion. EuroIntervention. (2018) 13:e2182–9. 10.4244/EIJ-D-17-00962 29400655

[37] Fujino A Mintz GS Lee T Hoshino M Usui E Kanaji Y. Predictors of calcium fracture derived from balloon angioplasty and its effect on stent expansion assessed by optical coherence tomography. JACC Cardiovasc Interv. (2018) 11:1015–7. 10.1016/j.jcin.2018.02.004 29798768

[38] van Ditzhuijzen NS Karanasos A Bruining N van den Heuvel M Sorop O Ligthart J. The impact of fourier-domain optical coherence tomography catheter induced motion artefacts on quantitative measurements of a PLLA-based bioresorbable scaffold. Int J Cardiovasc Imaging. (2014) 30:1013–26. 10.1007/s10554-014-0447-3 24831994

[39] Ino Y Kubo T Matsuo Y Yamaguchi T Shiono Y Shimamura K. Optical coherence tomography predictors for edge restenosis after everolimus-eluting stent implantation. Circula Cardiovasc Interv. (2016) 9:e004231. 10.1161/CIRCINTERVENTIONS.116.004231 27688261

[40] Prati F Romagnoli E Gatto L La Manna A Burzotta F Limbruno U. Clinical impact of suboptimal stenting and residual intrastent plaque/thrombus protrusion in patients with acute coronary syndrome. Circul Cardiovasc Interv. (2016) 9:e003726. 10.1161/CIRCINTERVENTIONS.115.003726 27965297

[41] Prati F Romagnoli E Manna A La Burzotta F Gatto L Marco V. Long-term consequences of optical coherence tomography findings during percutaneous coronary intervention: the centro per la lotta contro l'infarto – optimization of percutaneous coronary intervention (CLI-OPCI) LATE study. EuroIntervention. (2018) 14:e443–51. 10.4244/EIJ-D-17-01111 29633940

[42] Prati F Kodama T Romagnoli E Gatto L Di Vito L Ramazzotti V. Suboptimal stent deployment is associated with subacute stent thrombosis: optical coherence tomography insights from a multicenter matched study. From the CLI foundation investigators: the CLI-THRO study. Am Heart J. (2015) 169:249–56. 10.1016/j.ahj.2014.11.012 25641534

[43] Ha J Kim J-S Lim J Kim G Lee S Lee JS. Assessing computational fractional flow reserve from optical coherence tomography in patients with intermediate coronary stenosis in the left anterior descending artery. Circul Cardiovasc Intervent. (2016) 9:e003613. 10.1161/CIRCINTERVENTIONS.116.003613 27502209

[44] Burzotta F Leone AM Aurigemma C Zambrano A Zimbardo G Arioti M. Fractional flow reserve or optical coherence tomography to guide management of angiographically intermediate coronary stenosis: a single-center trial. JACC Cardiovasc Intervent. (2020) 13:49–58. 10.1016/j.jcin.2019.09.034 31918942

[45] Schneider VS Böhm F Blum K Riedel M Abdelwahed YS Klotsche J. Impact of real-time angiographic co-registered optical coherence tomography on percutaneous coronary intervention: the OPTICO-integration II trial. Clin Res Cardiol. (2021) 110:249–57. 10.1007/s00392-020-01739-1 32889633 PMC7862500

[46] Koyama K Fujino A Maehara A Yamamoto MH Alexandru D Jennings J. A prospective, single-center, randomized study to assess whether automated coregistration of optical coherence tomography with angiography can reduce geographic miss. Catheter Cardiovasc Interv. (2019) 93:411–8. 10.1002/ccd.27854 30345635

[47] Shlofmitz E Sosa F Goldberg A Maehara A Ali ZA Mintz GS. Bifurcation and ostial optical coherence tomography mapping (BOOM) - case description of a novel bifurcation stent technique. Cardiovasc Revasc Med. (2018) 19:47–9. 10.1016/j.carrev.2018.05.005 29779971

[48] Räber L Mintz GS Koskinas KC Johnson TW Holm NR Onuma Y. Clinical use of intracoronary imaging. Part 1: guidance and optimization of coronary interventions. An expert consensus document of the European association of percutaneous cardiovascular interventions. Eur Heart J. (2018) 39:3281–300. 10.1093/eurheartj/ehy285 29790954

[49] Doi H Maehara A Mintz GS Yu A Wang H Mandinov L. Impact of post-intervention minimal stent area on 9-month follow-up patency of paclitaxel-eluting stents: an integrated intravascular ultrasound analysis from the TAXUS IV, V, and VI and TAXUS ATLAS workhorse, long lesion, and direct stent trials. JACC Cardiovasc Interv. (2009) 2:1269–75. 10.1016/j.jcin.2009.10.005 20129555

[50] Soeda T Uemura S Park S-J Jang Y Lee S Cho J-M. Incidence and clinical significance of poststent optical coherence tomography findings: one-year follow-up study from a multicenter registry. Circulation. (2015) 132:1020–9. 10.1161/CIRCULATIONAHA.114.014704 26162917

[51] Fujii K Carlier SG Mintz GS Yang Y Moussa I Weisz G. Stent underexpansion and residual reference segment stenosis are related to stent thrombosis after sirolimus-eluting stent implantation: an intravascular ultrasound study. J Am Coll Cardiol. (2005) 45:995–8. 10.1016/j.jacc.2004.12.066 15808753

[52] Hong M-K Mintz GS Lee CW Park D-W Choi B-R Park K-H. Intravascular ultrasound predictors of angiographic restenosis after sirolimus-eluting stent implantation. Eur Heart J. (2006) 27:1305–10. 10.1093/eurheartj/ehi882 16682378

[53] Sonoda S Morino Y Ako J Terashima M Hassan AHM Bonneau HN. Impact of final stent dimensions on long-term results following sirolimus-eluting stent implantation: serial intravascular ultrasound analysis from the sirius trial. J Am Coll Cardiol. (2004) 43:1959–63. 10.1016/j.jacc.2004.01.044 15172398

[54] Morino Y Honda Y Okura H Oshima A Hayase M Bonneau HN. An optimal diagnostic threshold for minimal stent area to predict target lesion revascularization following stent implantation in native coronary lesions. Am J Cardiol. (2001) 88:301–3. 10.1016/S0002-9149(01)01646-0 11472713

[55] Gupta A Chhikara S Singh N Prasad K. Optical coherence tomography-guided management of underexpanded stent in calcified coronary lesion. BMJ Case Rep. (2021) 14:e239143. 10.1136/bcr-2020-239143 33495188 PMC7839892

[56] Wang B Mintz GS Witzenbichler B Souza CF Metzger DC Rinaldi MJ. Predictors and long-term clinical impact of acute stent malapposition: an assessment of dual antiplatelet therapy with drug-eluting stents (ADAPT-DES) intravascular ultrasound substudy. J Am Heart Assoc. (2021) 5:e004438. 10.1161/JAHA.116.004438 28007741 PMC5210413

[57] Guo N Maehara A Mintz GS He Y Xu K Wu X. Incidence, mechanisms, predictors, and clinical impact of acute and late stent malapposition after primary intervention in patients with acute myocardial infarction: an intravascular ultrasound substudy of the harmonizing outcomes with revascularization a. Circulation. (2010) 122:1077–84. 10.1161/CIRCULATIONAHA.109.906040 20805433

[58] Romagnoli E Gatto L La Manna A Burzotta F Taglieri N Saia F. Role of residual acute stent malapposition in percutaneous coronary interventions. Catheter Cardiovasc Interv. (2017) 90:566–75. 10.1002/ccd.26974 28295990

[59] Foin N Lu S Ng J Bulluck H Hausenloy DJ Wong PE. Stent malapposition and the risk of stent thrombosis: mechanistic insights from an in vitro model. EuroIntervention. (2017) 13:e1096–8. 10.4244/EIJ-D-17-00381 28781241

[60] Adriaenssens T Joner M Godschalk TC Malik N Alfonso F Xhepa E. Optical coherence tomography findings in patients with coronary stent thrombosis: a report of the PRESTIGE consortium (prevention of late stent thrombosis by an interdisciplinary global European effort). Circulation. (2017) 136:1007–21. 10.161/CIRCULATIONAHA.117.026788 28720725 PMC5598909

[61] Souteyrand G Amabile N Mangin L Chabin X Meneveau N Cayla G. Mechanisms of stent thrombosis analysed by optical coherence tomography: insights from the national PESTO french registry. Eur Heart J. (2016) 37:1208–16. 10.1093/eurheartj/ehv711 26757787

[62] Cuesta J Rivero F Bastante T García-Guimaraes M Antuna P Alvarado T. Optical coherence tomography findings in patients with stent thrombosis. Rev Española Cardiol. (2017) 70:1050–8. 10.1016/j.rec.2017.04.001 28495489

[63] Kawamori H Shite J Shinke T Otake H Matsumoto D Nakagawa M. Natural consequence of post-intervention stent malapposition, thrombus, tissue prolapse, and dissection assessed by optical coherence tomography at mid-term follow-up. Eur Heart J Cardiovasc Imaging. (2013) 14:865–75. 10.1093/ehjci/jes299 23291393 PMC3738096

[64] Sotomi Y Onuma Y Dijkstra J Miyazaki Y Kozuma K Tanabe K. Fate of post-procedural malapposition of everolimus-eluting polymeric bioresorbable scaffold and everolimus-eluting cobalt chromium metallic stent in human coronary arteries: sequential assessment with optical coherence tomography in ABSORB Japan trial. Eur Heart J Cardiovasc Imaging. (2018) 19:59–66. 10.1093/ehjci/jew329 28158421

[65] Chamié D Bezerra HG Attizzani GF Yamamoto H Kanaya T Stefano GT. Incidence, predictors, morphological characteristics, and clinical outcomes of stent edge dissections detected by optical coherence tomography. JACC Cardiovasc Interv. (2013) 6:800–13. 10.1016/j.jcin.2013.03.019 23871510

[66] van Zandvoort LJC Tomaniak M Tovar Forero MN Masdjedi K Visseren L Witberg K. Predictors for clinical outcome of untreated stent edge dissections as detected by optical coherence tomography. Circul Cardiovasc Interv. (2020) 13:e008685. 10.1161/CIRCINTERVENTIONS.119.008685 32089001

[67] Sugiyama T Kimura S Akiyama D Hishikari K Kawaguchi N Kamiishi T. Quantitative assessment of tissue prolapse on optical coherence tomography and its relation to underlying plaque morphologies and clinical outcome in patients with elective stent implantation. Int J Cardiol. (2014) 176:182–90. 10.1016/j.ijcard.2014.07.005 25042663

[68] Hong YJ Jeong MH Choi YH Song JA Kim DH Lee KH. Impact of tissue prolapse after stent implantation on short-and long-term clinical outcomes in patients with acute myocardial infarction: an intravascular ultrasound analysis. Int J Cardiol. (2013) 166:646–51. 10.1016/j.ijcard.2011.11.092 22177591

[69] Choi S-Y Witzenbichler B Maehara A Lansky AJ Guagliumi G Brodie B. Intravascular ultrasound findings of early stent thrombosis after primary percutaneous intervention in acute myocardial infarction: a harmonizing outcomes with revascularization and stents in acute myocardial infarction (HORIZONS-AMI) substudy. Circul Cardiovasc Interv. (2011) 4:239–47. 10.1161/CIRCINTERVENTIONS.110.959791 21586693

[70] Johnson TW Räber L Mario C Di Bourantas C V Jia H Mattesini A. Clinical use of intracoronary imaging. Part 2: acute coronary syndromes, ambiguous coronary angiography findings, and guiding interventional decision-making: an expert consensus document of the European association of percutaneous cardiovascular intervent. EuroIntervention. (2019) 15:434–51. 10.4244/EIJY19M06_02 31258132

[71] Jia H Abtahian F Aguirre AD Lee S Chia S Lowe H. In vivo diagnosis of plaque erosion and calcified nodule in patients with acute coronary syndrome by intravascular optical coherence tomography. J Am Coll Cardiol. (2013) 62:1748–58. 10.1016/j.jacc.2013.05.071 23810884 PMC3874870

[72] Kramer MCA Rittersma SZH de Winter RJ Ladich ER Fowler DR Liang Y-H. Relationship of thrombus healing to underlying plaque morphology in sudden coronary death. J Am Coll Cardiol. (2010) 55:122–32. 10.1016/j.jacc.2009.09.007 19818571

[73] Xing L Yamamoto E Sugiyama T Jia H Ma L Hu S. EROSION study (effective anti-thrombotic therapy without stenting: intravascular optical coherence tomography–based management in plaque erosion). Circulation Cardiovasc Interv. (2017) 10:e005860. 10.1161/CIRCINTERVENTIONS.117.005860 29246916

[74] Sugiyama T Yamamoto E Fracassi F Lee H Yonetsu T Kakuta T. Calcified plaques in patients with acute coronary syndromes. JACC Cardiovasc Interv. (2019) 12:531–40. 10.1016/j.jcin.2018.12.013 30898249

[75] Adlam D Alfonso F Maas A Vrints C. European society of cardiology, acute cardiovascular care association, SCAD study group: a position paper on spontaneous coronary artery dissection. Eur Heart J. (2018) 39:3353–68. 10.1093/eurheartj/ehy080 29481627 PMC6148526

[76] Nakazawa G Otsuka F Nakano M Vorpahl M Yazdani SK Ladich E. The pathology of neoatherosclerosis in human coronary implants bare-metal and drug-eluting stents. J Am Coll Cardiol. (2011) 57:1314–22. 10.1016/j.jacc.2011.01.011 21376502 PMC3093310

[77] Habara M Terashima M Nasu K Kaneda H Inoue K Ito T. Difference of tissue characteristics between early and very late restenosis lesions after bare-metal stent implantation: an optical coherence tomography study. Circul Cardiovasc Interv. (2011) 4:232–8. 10.1161/CIRCINTERVENTIONS.110.959999 21610225

[78] Sethi A Malhotra G Singh S Singh PP Khosla S. Efficacy of various percutaneous interventions for in-stent restenosis: comprehensive network meta-analysis of randomized controlled trials. Circul Cardiovasc Interv. (2015) 8:e002778. 10.1161/CIRCINTERVENTIONS.115.002778 26546577

[79] Giacoppo D Alfonso F Xu B Claessen BEPM Adriaenssens T Jensen C. drug-coated balloon angioplasty versus drug-eluting stent implantation in patients with coronary stent restenosis. J Am Coll Cardiol. (2020) 75:2664–78. 10.1016/j.jacc.2020.04.006 32466881

[80] Alfonso F Pérez-Vizcayno MJ Del Blanco BG García-Touchard A Masotti M López-Minguez JR. Comparison of the efficacy of everolimus-eluting stents versus drug-eluting balloons in patients with in-stent restenosis (from the RIBS IV and V randomized clinical trials). Am J Cardiol. (2016) 117:546–54. 10.1016/j.amjcard.2015.11.042 26725102

[81] Stefanini GG Kalesan B Serruys PW Heg D Buszman P Linke A. Long-term clinical outcomes of biodegradable polymer biolimus-eluting stents versus durable polymer sirolimus-eluting stents in patients with coronary artery disease (LEADERS): 4 year follow-up of a randomised non-inferiority trial. Lancet. (2011) 378:1940–8. 10.1016/S0140-6736(11)61672-3 22075451

[82] Chen BX Ma FY Luo W Ruan JH Xie WL Zhao XZ. Neointimal coverage of bare-metal and sirolimus-eluting stents evaluated with optical coherence tomography. Heart. (2008) 94:566–70. 10.1136/hrt.2007.118679 17923466 PMC2564839

[83] Liang M Tan H-C Low AF. Three-year follow-up optical coherence tomography of under-expanded drug-eluting stent in-stent restenosis treated with ABSORB bioresorbable vascular scaffold following ultra-high pressure pre-dilatation. J Cardiol cases. (2017) 17:4–8. 10.1016/j.jccase.2017.08.015 30279842 PMC6146415

[84] Woudstra P Grundeken MJ Kraak RP Hassell MECJ Arkenbout EK Baan JJ. Amsterdam investigator-initiateD absorb strategy all-comers trial (AIDA trial): a clinical evaluation comparing the efficacy and performance of ABSORB everolimus-eluting bioresorbable vascular scaffold strategy vs the XIENCE family XIENCE PRIME or XIENC. Am Heart J. (2014) 167:133–40. 10.1016/j.ahj.2013.09.017 24439973

[85] Moscarella E Ielasi A Granata F Coscarelli S Stabile E Latib A. Long-term clinical outcomes after bioresorbable vascular scaffold implantation for the treatment of coronary in-stent restenosis: a multicenter Italian experience. Circul Cardiovasc Interv. (2016) 9:e003148. 10.1161/CIRCINTERVENTIONS.115.003148 27059683

[86] Ellis SG Kereiakes DJ Metzger DC Caputo RP Rizik DG Teirstein PS. Everolimus-eluting bioresorbable scaffolds for coronary artery disease. N Engl J Med. (2015) 373:1905–15. 10.1056/NEJMoa1509038 26457558

[87] Kereiakes DJ Ellis SG Metzger C Caputo RP Rizik DG Teirstein PS. 3-year clinical outcomes with everolimus-eluting bioresorbable coronary scaffolds: the ABSORB III trial. J Am Coll Cardiol. (2017) 70:2852–62. 10.1016/j.jacc.2017.10.010 29100702

[88] Serruys PW Chevalier B Sotomi Y Cequier A Carrié D Piek JJ. Comparison of an everolimus-eluting bioresorbable scaffold with an everolimus-eluting metallic stent for the treatment of coronary artery stenosis (ABSORB II): a 3 year, randomised, controlled, single-blind, multicentre clinical trial. The Lancet. (2016) 388:2479–91. 10.1016/S0140-6736(16)32050-5 27806897

[89] Ali ZA Gao R Kimura T Onuma Y Kereiakes DJ Ellis SG. Three-year outcomes with the absorb bioresorbable scaffold: individual-patient-data meta-analysis from the ABSORB randomized trials. Circulation. (2018) 137:464–79. 10.1161/CIRCULATIONAHA.117.031843 29089314

[90] Kereiakes DJ Ellis SG Metzger DC Caputo RP Rizik DG Teirstein PS. Clinical outcomes before and after complete everolimus-eluting bioresorbable scaffold resorption. Circulation. (2019) 140:1895–903. 10.1161/CIRCULATIONAHA.119.042584 31553222

[91] Polimeni A Weissner M Schochlow K Ullrich H Indolfi C Dijkstra J. Incidence, clinical presentation, and predictors of clinical restenosis in coronary bioresorbable scaffolds. JACC Cardiovasc Interv. (2017) 10:1819–27. 10.1016/j.jcin.2017.07.034 28935073

[92] Koppara T Cheng Q Yahagi K Mori H Sanchez OD Feygin J. Thrombogenicity and early vascular healing response in metallic biodegradable polymer-based and fully bioabsorbable drug-eluting stents. Circul Cardiovasc Interv. (2015) 8:e002427. 10.1161/CIRCINTERVENTIONS.115.002427 26022535

[93] Lassen JF Holm NR Stankovic G Lefèvre T Chieffo A Hildick-Smith D. Percutaneous coronary intervention for coronary bifurcation disease: consensus from the first 10 years of the European bifurcation club meetings. EuroIntervention. (2014) 10:545–60. 10.4244/EIJV10I5A97 25256198

[94] Watanabe M Uemura S Sugawara Y Ueda T Soeda T Takeda Y. Side branch complication after a single-stent crossover technique: prediction with frequency domain optical coherence tomography. Coron Artery Dis. (2014) 25:321–9. 10.1097/MCA.0000000000000091 24769514

[95] Okamura T Onuma Y Yamada J Iqbal J Tateishi H Nao T. 3D optical coherence tomography: new insights into the process of optimal rewiring of side branches during bifurcational stenting. EuroIntervention. (2014) 10:907–15. 10.4244/EIJV10I8A157 24531393

[96] Karanasos A Tu S van Ditzhuijzen NS Ligthart JMR Witberg K Van Mieghem N. A novel method to assess coronary artery bifurcations by OCT: cut-plane analysis for side-branch ostial assessment from a main-vessel pullback. Euro Heart J Cardiovasc Imaging. (2015) 16:177–89. 10.1093/ehjci/jeu176 25227268

[97] Burzotta F Dato I Trani C Pirozzolo G De Maria GL Porto I. Frequency domain optical coherence tomography to assess non-ostial left main coronary artery. EuroIntervention. (2015) 10:e1–8. 10.4244/EIJV10I9A179 25599698

[98] Fujino Y Bezerra HG Attizzani GF Wang W Yamamoto H Chamié D. Frequency-domain optical coherence tomography assessment of unprotected left main coronary artery disease-a comparison with intravascular ultrasound. Catheter Cardiovasc Interv. (2013) 82:E173–83. 10.1002/ccd.24843 23359350

[99] Cortese B Burzotta F Alfonso F Pellegrini D Trani C Aurigemma C. Role of optical coherence tomography for distal left main stem angioplasty. Catheter Cardiovasc Interv. (2020) 96:755–61. 10.1002/ccd.28547 31631525

[100] Amabile N Rangé G Souteyrand G Godin M Boussaada MM Meneveau N. Optical coherence tomography to guide percutaneous coronary intervention of the left main coronary artery: the LEMON study. EuroIntervention. (2021) 17:e124–31. 10.4244/EIJ-D-20-01121 33226003 PMC9724912

[101] Gupta A Datta R Chhikara S Dhagat PK Vijayvergiya R. Coronary artery aneurysm after drug-eluting stent implantation causing coronary-bronchial fistula. JACC Case Rep. (2020) 2:1692–7. 10.1016/j.jaccas.2020.07.041 34317036 PMC8312138

[102] Gupta A Chhikara S Datta R Vijayvergiya R. Everolimus-Eluting stent causing coronary artery aneurysm in 7 days: 3D-OCT findings and management. J Invasive Cardiol. (2020) 32:E301–2. 33130600 10.25270/jic/20.00025

[103] Ozaki Y Kitabata H Tsujioka H Hosokawa S Kashiwagi M Ishibashi K. Comparison of contrast media and low-molecular-weight dextran for frequency-domain optical coherence tomography. Circul J. (2012) 76:922–7. 10.1253/circj.CJ-11-1122 22301848

[104] Vijayvergiya R Ratheesh KJ Gupta A. Low molecular weight dextran: an alternative to radiographic contrast agent for optical coherence tomography imaging. IHJ Cardiovasc Case Rep. (2017) 1:10–1. 10.1016/j.ihjccr.2017.03.002

[105] Kurogi K Ishii M Sakamoto K Komaki S Kusaka H Yamamoto N. Optical coherence tomography-guided percutaneous coronary intervention with low-molecular-weight dextran— effect on renal function. Circul J. (2020) 84:917–25. 10.1253/circj.CJ-20-0093 32350234

[106] Seeliger E Flemming B Wronski T Ladwig M Arakelyan K Godes M. Viscosity of contrast media perturbs renal hemodynamics. J Am Soc Nephrol. (2007) 18:2912–20. 10.1681/ASN.2006111216 17942967

[107] Mahesh NK Gupta A Barward P Vijayvergiya R Sharma P Mahesh A. Study of saline optical coherence tomography-guided percutaneous coronary intervention (SOCT-PCI study). Indian Heart J. (2020) 72:239–43. 10.1016/j.ihj.2020.03.013 32861376 PMC7474129

[108] Gupta A Chhikara S Vijayvergiya R Seth A Mahesh NK Akasaka T. Saline as an alternative to radio-contrast for optical coherence tomography guided percutaneous coronary intervention: a prospective comparison. Cardiovasc Revasc Med. (2021) 34:86–91. 10.1016/j.carrev.2021.01.010 33468422

[109] Chu M Jia H Gutiérrez-Chico JL Maehara A Ali ZA Zeng X. Artificial intelligence and optical coherence tomography for the automatic characterisation of human atherosclerotic plaques. EuroIntervention. (2021) 17:41–50. 10.4244/EIJ-D-20-01355 33528359 PMC9724931

[110] Sheth TN Pinilla-Echeverri N Mehta SR Courtney BK. First-in-human images of coronary atherosclerosis and coronary stents using a novel hybrid intravascular ultrasound and optical coherence tomographic catheter. JACC Cardiovasc Interv. (2018) 11:2427–30. 10.1016/j.jcin.2018.09.022 30522674

